# Supplementary material for: Substantially Altered Expression Profile of Diabetes/Cardiovascular/Cerebrovascular Disease Associated microRNAs in Children Descending from Pregnancy Complicated by Gestational Diabetes Mellitus—One of Several Possible Reasons for an Increased Cardiovascular Risk
Source: Cells. 2020 Jun 26;9(6):1557. doi: 10.3390/cells9061557 (PMC7349356; doi:10.3390/cells9061557)
Supplement: Supplementary file 1 [file cells-09-01557-s001.pdf]

Supplementary Material

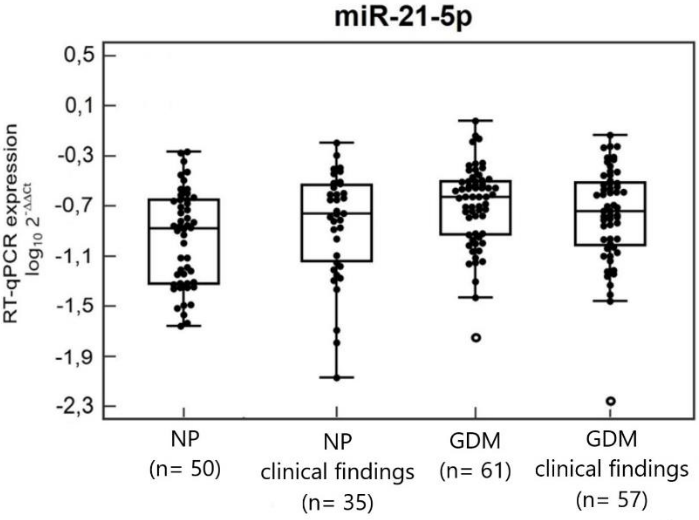

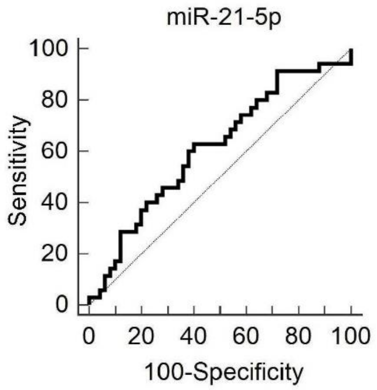

Area under the ROC curve (AUC)	0.613
Standard Error	0.0627
95% Confidence interval	0.501 to 0.716
Significance level P (Area=0.5)	0.0727

Estimated sensitivity at fixed specificity			
Specificity	Sensitivity	95% CI	Criterion
90.00	17.14	0.00 to 40.00	>0.323192755

Criterion	Sensitivity	95% CI	Specificity	95% CI	+LR	95% CI	-LR	95% CI
>0.149341833	62.86	44.9 - 78.5	60.00	45.2 - 73.6	1.57	1.0 - 2.4	0.62	0.4 - 1.0

Supplementary Figure S1: Aberrant microRNA expression profile in children descending from normal pregnancies. A trend towards up-regulation of miR-21-5p was observed in children with abnormal clinical findings when the comparison to children with normal clinical findings was performed. NP, normal pregnancies; GDM, gestational diabetes mellitus.

A

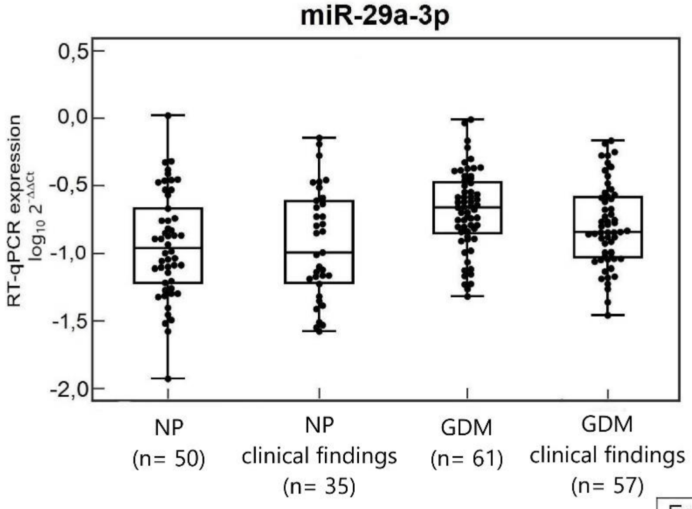

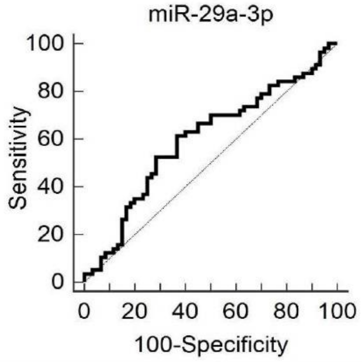

Area under the ROC curve (AUC)	0.603
Standard Error	0.0530
95% Confidence interval	0.509 to 0.692
Significance level P (Area=0.5)	0.0524

Estimated sensitivity at fixed specificity			
Specificity	Sensitivity	95% CI	Criterion
90.00	12.28	1.75 to 33.33	≤0.069056424

Criterion	Sensitivity	95% CI	Specificity	95% CI	+LR	95% CI	-LR	95% CI
≤0.171819529	61.40	47.6 - 74.0	63.93	50.6 - 75.8	1.70	1.1 - 2.5	0.60	0.4 - 0.9

B

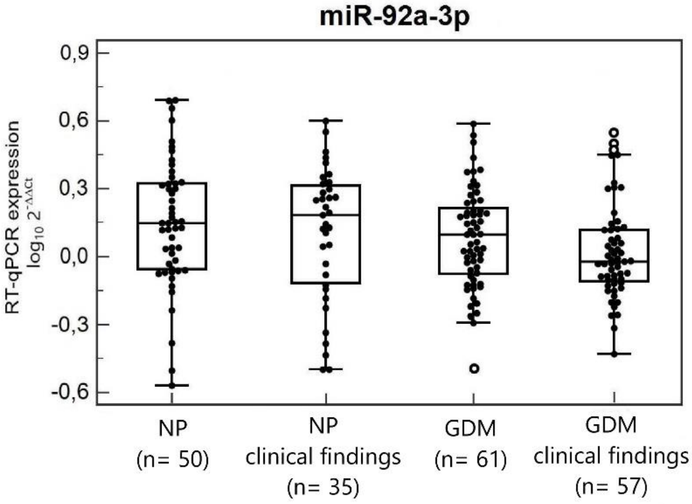

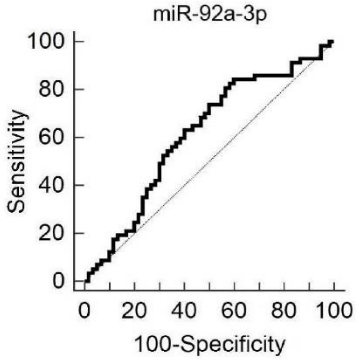

Area under the ROC curve (AUC)	0.616
Standard Error	0.0525
95% Confidence interval	0.522 to 0.704
Significance level P (Area=0.5)	0.0268

Estimated sensitivity at fixed specificity			
Specificity	Sensitivity	95% CI	Criterion
90.00	12.28	1.75 to 26.32	≤0.626507233

Criterion	Sensitivity	95% CI	Specificity	95% CI	+LR	95% CI	-LR	95% CI
≤1.381704532	82.46	70.1 - 91.3	42.62	30.0 - 55.9	1.44	1.1 - 1.8	0.41	0.2 - 0.8

Supplementary Figure S2: Aberrant microRNA expression profile in children descending from GDM complicated pregnancies. (**A**,**B**) Down-regulation of miR-29a-3p and miR-92a-3p was observed in children with abnormal clinical findings when the comparison to children with normal clinical findings was performed. NP, normal pregnancies; GDM, gestational diabetes mellitus.

A

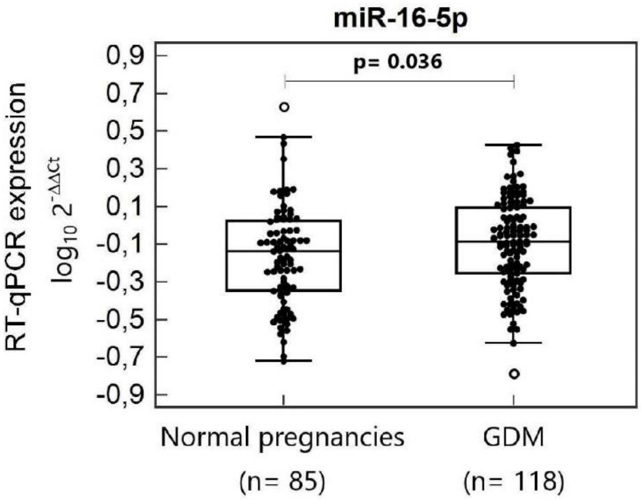

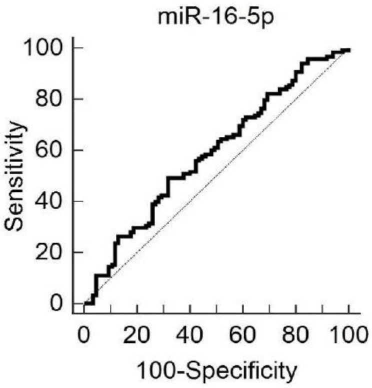

Area under the ROC curve (AUC)	0.586
Standard Error	0.0407
95% Confidence interval	0.515 to 0.655
Significance level P (Area=0.5)	0.0342

Estimated sensitivity at fixed specificity			
Specificity	Sensitivity	95% CI	Criterion
90.00	14.41	5.93 to 31.36	>1.451537049

Criterion	Sensitivity	95% CI	Specificity	95% CI	+LR	95% CI	-LR	95% CI
>0.846738909	49.15	39.8 - 58.5	68.24	57.2 - 77.9	1.55	1.1 - 2.2	0.75	0.6 - 0.9

B

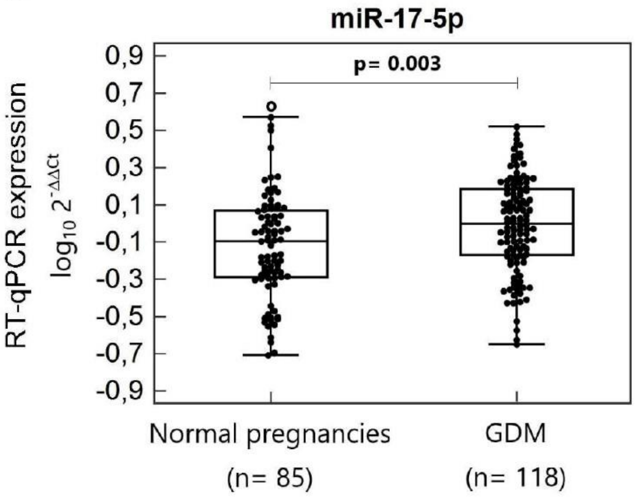

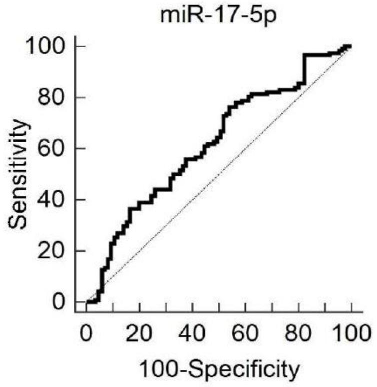

Area under the ROC curve (AUC)	0.620
Standard Error	0.0400
95% Confidence interval	0.550 to 0.687
Significance level P (Area=0.5)	0.0026

Estimated sensitivity at fixed specificity			
Specificity	Sensitivity	95% CI	Criterion
90.00	22.88	2.54 to 37.29	>1.54241536

Criterion	Sensitivity	95% CI	Specificity	95% CI	+LR	95% CI	-LR	95% CI
>0.66215296	76.27	67.6 - 83.6	45.88	35.0 - 57.0	1.41	1.1 - 1.8	0.52	0.3 - 0.8

C

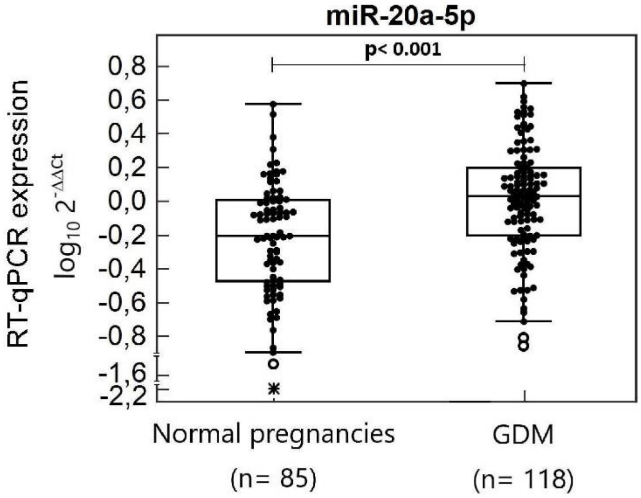

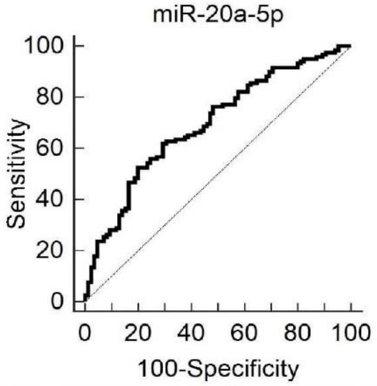

Area under the ROC curve (AUC)	0.693
Standard Error	0.0372
95% Confidence interval	0.625 to 0.756
Significance level P (Area=0.5)	<0.0001

Estimated sensitivity at fixed specificity			
Specificity	Sensitivity	95% CI	Criterion
90.00	27.97	18.64 to 47.46	>1.4334805

Criterion	Sensitivity	95% CI	Specificity	95% CI	+LR	95% CI	-LR	95% CI
>1.042907	52.54	43.1 - 61.8	80.00	69.9 - 87.9	2.63	1.7 - 4.2	0.59	0.5 - 0.7

D

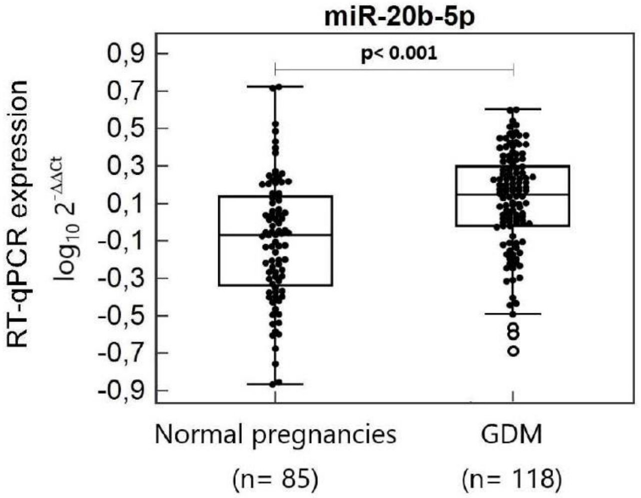

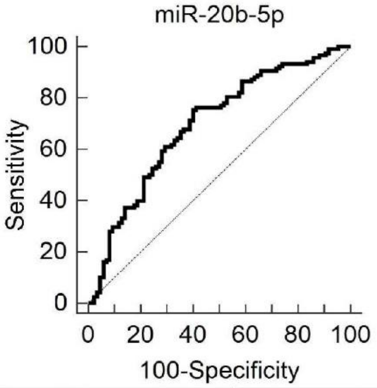

Area under the ROC curve (AUC)	0.695
Standard Error	0.0380
95% Confidence interval	0.626 to 0.757
Significance level P (Area=0.5)	<0.0001

Estimated sensitivity at fixed specificity			
Specificity	Sensitivity	95% CI	Criterion
90.00	29.66	11.02 to 44.92	>1.81441

Criterion	Sensitivity	95% CI	Specificity	95% CI	+LR	95% CI	-LR	95% CI
>0.953545	75.42	66.6 - 82.9	60.00	48.8 - 70.5	1.89	1.4 - 2.5	0.41	0.3 - 0.6

E

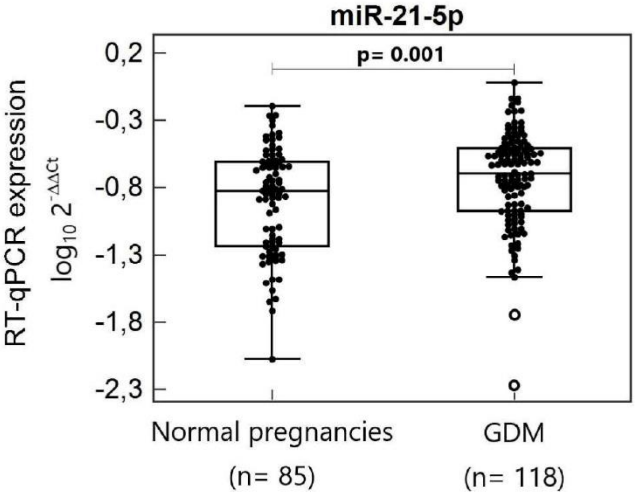

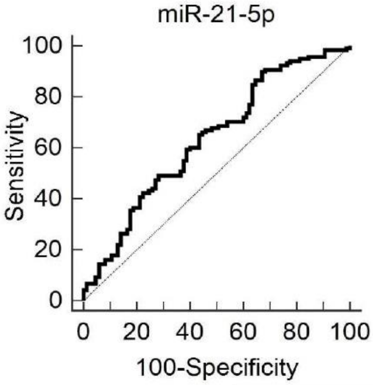

Area under the ROC curve (AUC)	0.633
Standard Error	0.0398
95% Confidence interval	0.563 to 0.700
Significance level P (Area=0.5)	0.0008

Estimated sensitivity at fixed specificity			
Specificity	Sensitivity	95% CI	Criterion
90.00	16.10	5.45 to 29.66	>0.37185669

Criterion	Sensitivity	95% CI	Specificity	95% CI	+LR	95% CI	-LR	95% CI
>0.06981769	89.83	82.9 - 94.6	32.94	23.1 - 44.0	1.34	1.1 - 1.6	0.31	0.2 - 0.6

F

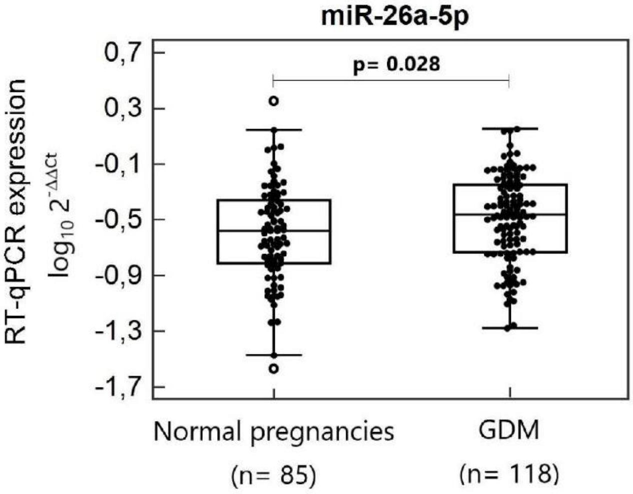

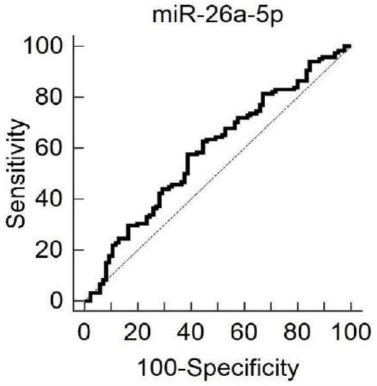

Area under the ROC curve (AUC)	0.591
Standard Error	0.0406
95% Confidence interval	0.520 to 0.659
Significance level P (Area=0.5)	0.0259

Estimated sensitivity at fixed specificity			
Specificity	Sensitivity	95% CI	Criterion
90.00	17.80	1.69 to 30.51	>0.6500598

Criterion	Sensitivity	95% CI	Specificity	95% CI	+LR	95% CI	-LR	95% CI
>0.314884334	57.63	48.2 - 66.7	61.18	50.0 - 71.6	1.48	1.1 - 2.0	0.69	0.5 - 0.9

G

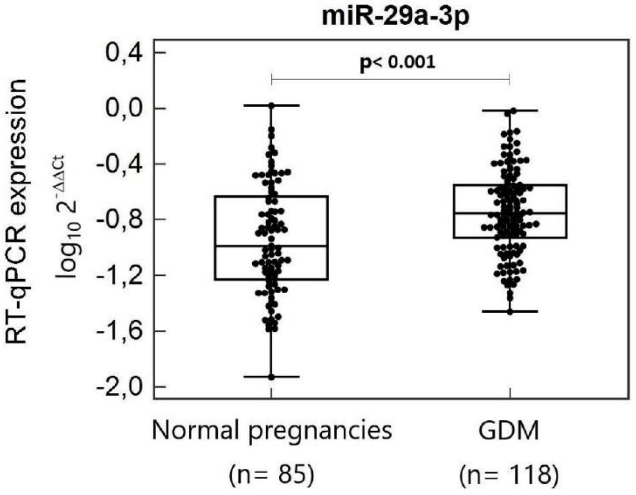

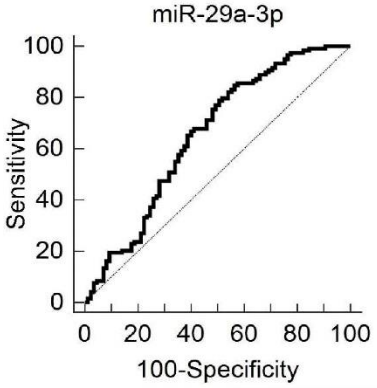

Area under the ROC curve (AUC)	0.653
Standard Error	0.0404
95% Confidence interval	0.583 to 0.718
Significance level P (Area=0.5)	0.0002

Estimated sensitivity at fixed specificity			
Specificity	Sensitivity	95% CI	Criterion
90.00	19.49	11.02 to 32.84	>0.349486706

Criterion	Sensitivity	95% CI	Specificity	95% CI	+LR	95% CI	-LR	95% CI
>0.086833444	84.75	77.0 - 90.7	43.53	32.8 - 54.7	1.50	1.2 - 1.8	0.35	0.2 - 0.6

H

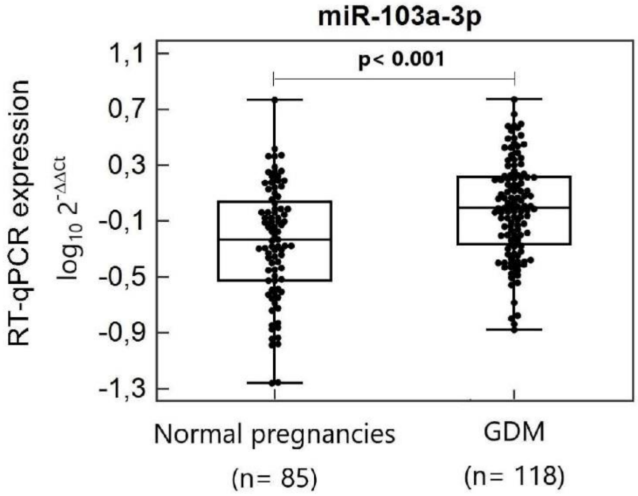

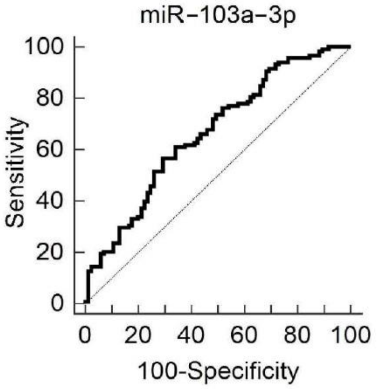

Area under the ROC curve (AUC)	0.665
Standard Error	0.0386
95% Confidence interval	0.596 to 0.730
Significance level P (Area=0.5)	<0.0001

Estimated sensitivity at fixed specificity			
Specificity	Sensitivity	95% CI	Criterion
90.00	20.34	7.63 to 31.36	>1.755066833

Criterion	Sensitivity	95% CI	Specificity	95% CI	+LR	95% CI	-LR	95% CI
>0.912860441	56.78	47.3 - 65.9	70.59	59.7 - 80.0	1.93	1.3 - 2.8	0.61	0.5 - 0.8

I

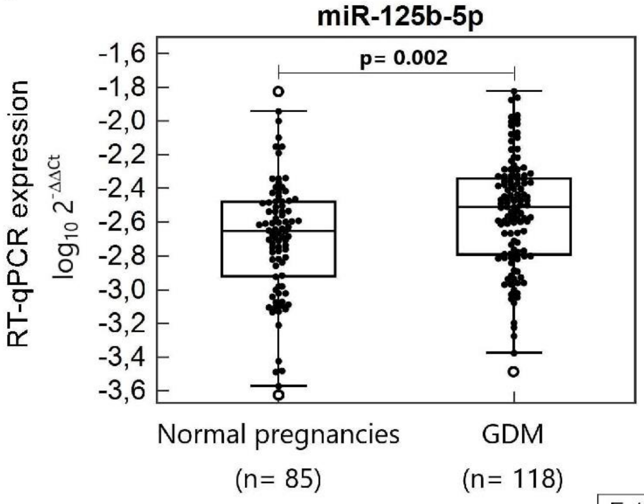

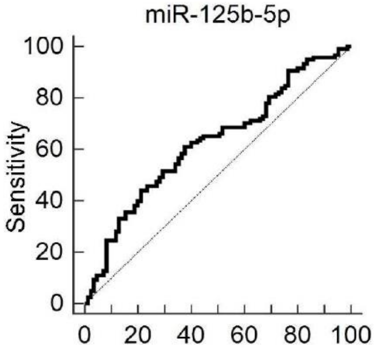

Area under the ROC curve (AUC)	0.626
Standard Error	0.0395
95% Confidence interval	0.556 to 0.693
Significance level P (Area=0.5)	0.0014

Estimated sensitivity at fixed specificity			
Specificity	Sensitivity	95% CI	Criterion
90.00	24.58	8.47 to 41.53	>0.004580526

Criterion	Sensitivity	95% CI	Specificity	95% CI	+LR	95% CI	-LR	95% CI
>0.002551344	61.02	51.6 - 69.9	62.35	51.2 - 72.6	1.62	1.2 - 2.2	0.63	0.5 - 0.8

J

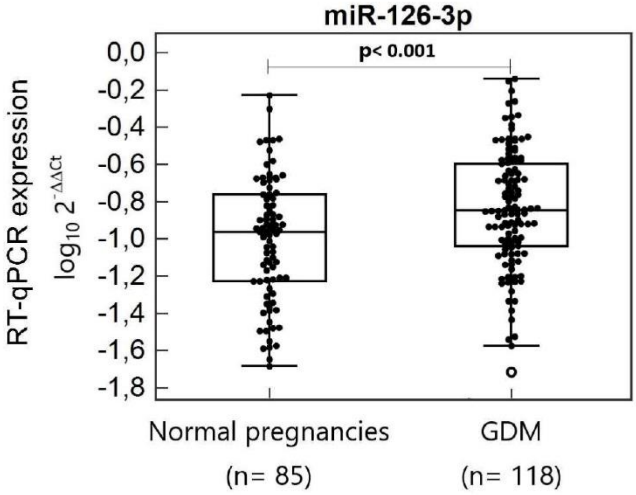

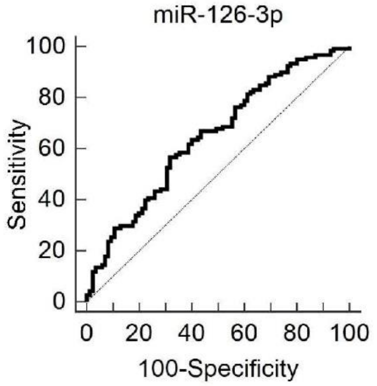

Area under the ROC curve (AUC)	0.646
Standard Error	0.0391
95% Confidence interval	0.576 to 0.712
Significance level P (Area=0.5)	0.0002

Estimated sensitivity at fixed specificity			
Specificity	Sensitivity	95% CI	Criterion
90.00	25.42	11.02 to 36.44	>0.242719505

Criterion	Sensitivity	95% CI	Specificity	95% CI	+LR	95% CI	-LR	95% CI
>0.134154053	56.78	47.3 - 65.9	68.24	57.2 - 77.9	1.79	1.3 - 2.5	0.63	0.5 - 0.8

K

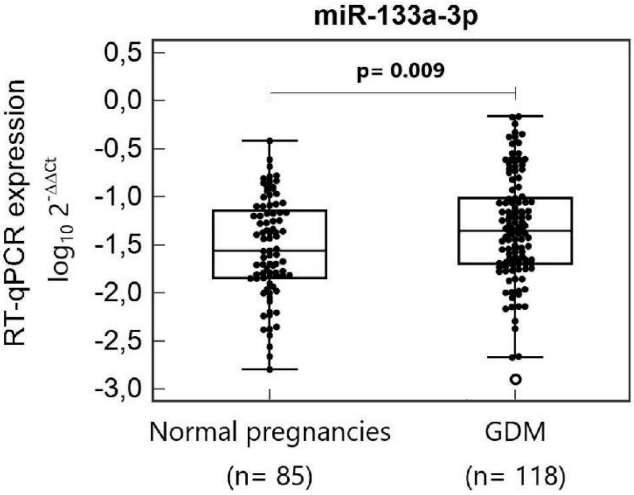

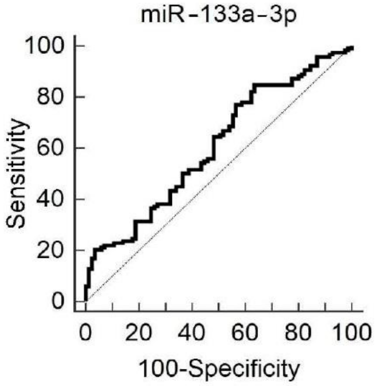

Area under the ROC curve (AUC)	0.607
Standard Error	0.0401
95% Confidence interval	0.536 to 0.674
Significance level P (Area=0.5)	0.0078

Estimated sensitivity at fixed specificity			
Specificity	Sensitivity	95% CI	Criterion
90.00	22.03	15.25 to 30.57	>0.132025307

Criterion	Sensitivity	95% CI	Specificity	95% CI	+LR	95% CI	-LR	95% CI
>0.016509996	84.75	77.0 - 90.7	36.47	26.3 - 47.6	1.33	1.1 - 1.6	0.42	0.3 - 0.7

L

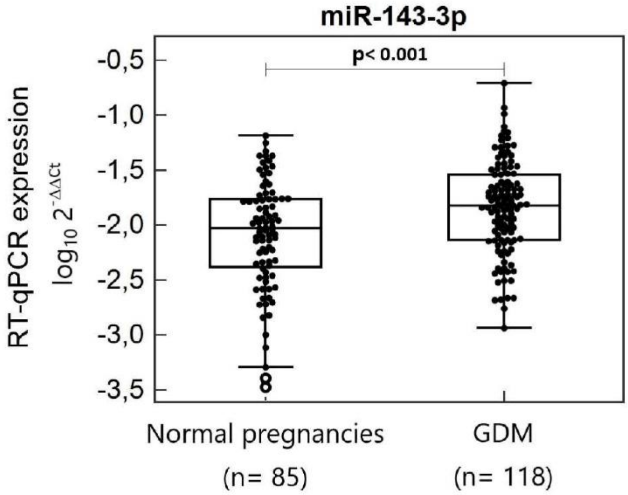

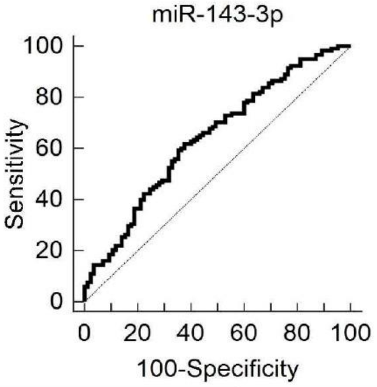

Area under the ROC curve (AUC)	0.641
Standard Error	0.0393
95% Confidence interval	0.571 to 0.707
Significance level P (Area=0.5)	0.0003

Estimated sensitivity at fixed specificity			
Specificity	Sensitivity	95% CI	Criterion
90.00	18.64	11.02 to 32.20	>0.033853987

Criterion	Sensitivity	95% CI	Specificity	95% CI	+LR	95% CI	-LR	95% CI
>0.011820045	61.86	52.5 - 70.6	62.35	51.2 - 72.6	1.64	1.2 - 2.2	0.61	0.5 - 0.8

M

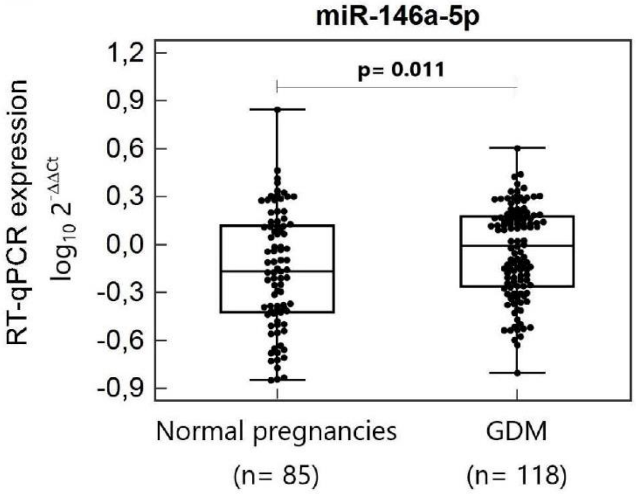

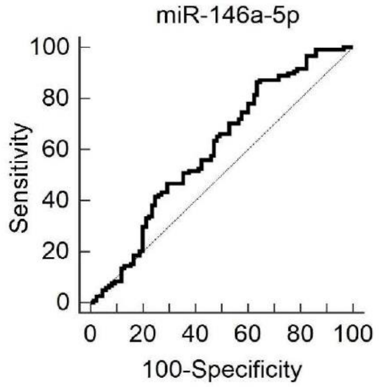

Area under the ROC curve (AUC)	0.604
Standard Error	0.0414
95% Confidence interval	0.534 to 0.672
Significance level P (Area=0.5)	0.0117

Estimated sensitivity at fixed specificity			
Specificity	Sensitivity	95% CI	Criterion
90.00	8.47	2.54 to 20.34	>1.977850467

Criterion	Sensitivity	95% CI	Specificity	95% CI	+LR	95% CI	-LR	95% CI
>0.421913076	86.44	78.9 - 92.0	36.47	26.3 - 47.6	1.36	1.1 - 1.6	0.37	0.2 - 0.6

N

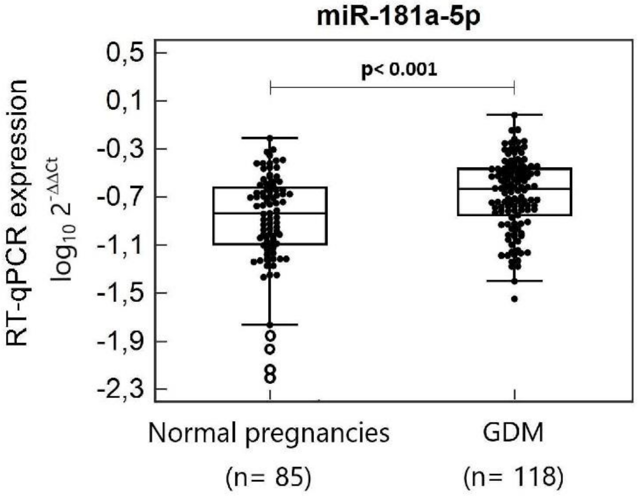

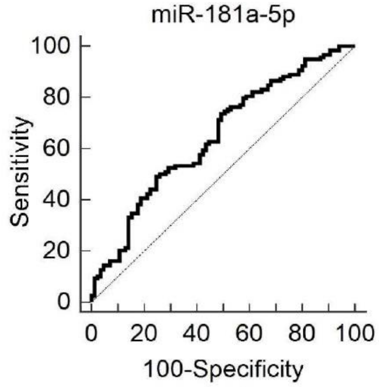

Area under the ROC curve (AUC)	0.647
Standard Error	0.0392
95% Confidence interval	0.577 to 0.712
Significance level P (Area=0.5)	0.0002

Estimated sensitivity at fixed specificity			
Specificity	Sensitivity	95% CI	Criterion
90.00	16.10	5.93 to 28.70	>0.378978361

Criterion	Sensitivity	95% CI	Specificity	95% CI	+LR	95% CI	-LR	95% CI
>0.234382158	49.15	39.8 - 58.5	75.29	64.7 - 84.0	1.99	1.3 - 3.0	0.68	0.5 - 0.8

O

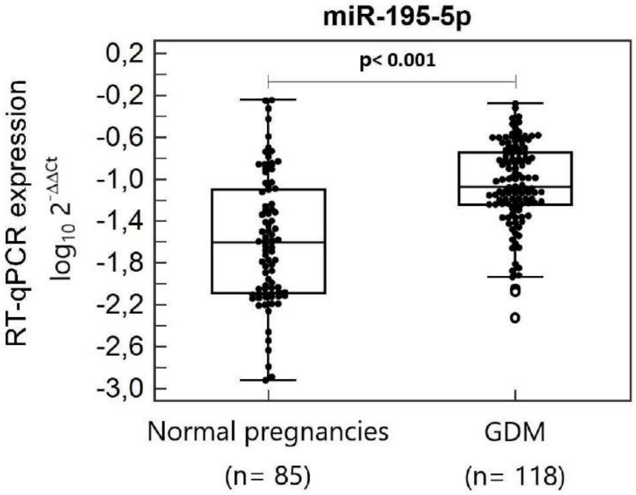

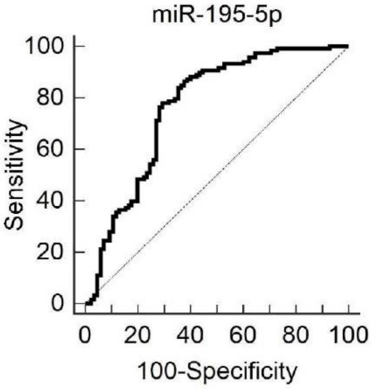

Area under the ROC curve (AUC)	0.765
Standard Error	0.0361
95% Confidence interval	0.700 to 0.821
Significance level P (Area=0.5)	<0.0001

Estimated sensitivity at fixed specificity			
Specificity	Sensitivity	95% CI	Criterion
90.00	27.97	2.54 to 41.53	>0.162032225

Criterion	Sensitivity	95% CI	Specificity	95% CI	+LR	95% CI	-LR	95% CI
>0.036219298	86.44	78.9 - 92.0	62.35	51.2 - 72.6	2.30	1.7 - 3.0	0.22	0.1 - 0.4

P

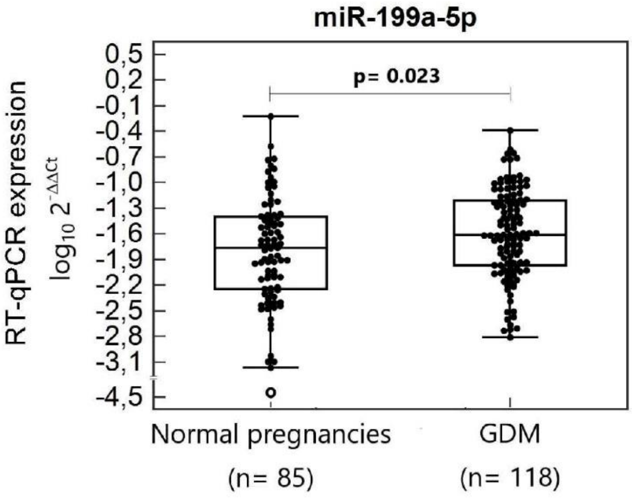

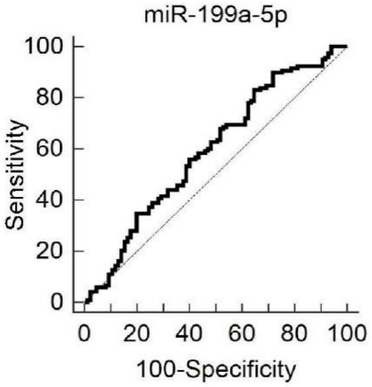

Area under the ROC curve (AUC)	0.594
Standard Error	0.0410
95% Confidence interval	0.523 to 0.662
Significance level P (Area=0.5)	0.0223

Estimated sensitivity at fixed specificity			
Specificity	Sensitivity	95% CI	Criterion
90.00	11.02	3.39 to 28.81	>0.101728811

Criterion	Sensitivity	95% CI	Specificity	95% CI	+LR	95% CI	-LR	95% CI
>0.008308903	83.05	75.0 - 89.3	35.29	25.2 - 46.4	1.28	1.1 - 1.5	0.48	0.3 - 0.8

Q

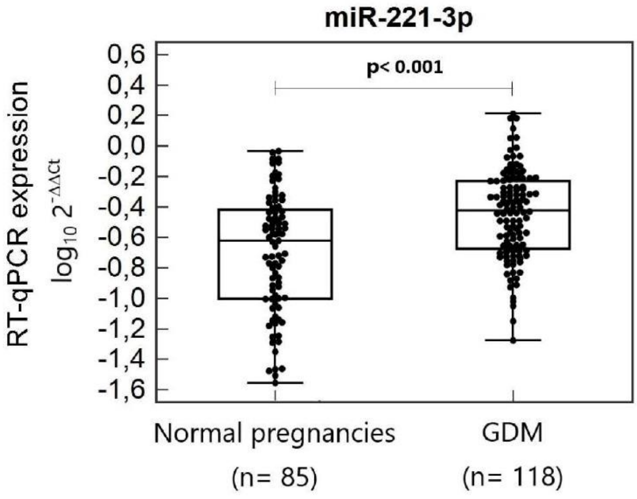

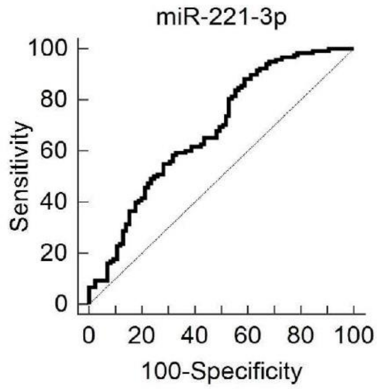

Area under the ROC curve (AUC)	0.683
Standard Error	0.0385
95% Confidence interval	0.614 to 0.746
Significance level P (Area=0.5)	<0.0001

Estimated sensitivity at fixed specificity			
Specificity	Sensitivity	95% CI	Criterion
90.00	17.80	4.17 to 35.56	>0.630703134

Criterion	Sensitivity	95% CI	Specificity	95% CI	+LR	95% CI	-LR	95% CI
>0.16975623	88.14	80.9 - 93.4	41.18	30.6 - 52.4	1.50	1.2 - 1.8	0.29	0.2 - 0.5

R

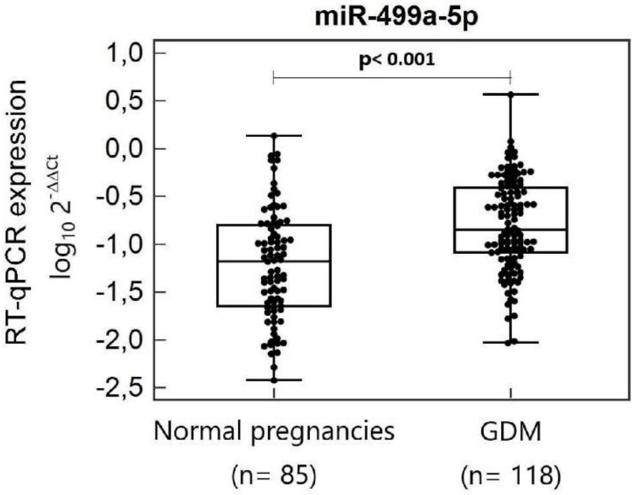

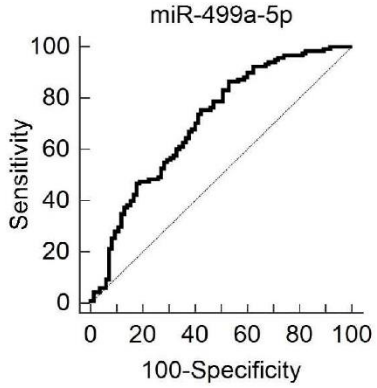

Area under the ROC curve (AUC)	0.709
Standard Error	0.0375
95% Confidence interval	0.641 to 0.770
Significance level P (Area=0.5)	<0.0001

Estimated sensitivity at fixed specificity			
Specificity	Sensitivity	95% CI	Criterion
90.00	27.97	5.08 to 44.92	>0.337048676

Criterion	Sensitivity	95% CI	Specificity	95% CI	+LR	95% CI	-LR	95% CI
>0.04701734	86.44	78.9 - 92.0	47.06	36.1 - 58.2	1.63	1.3 - 2.0	0.29	0.2 - 0.5

S

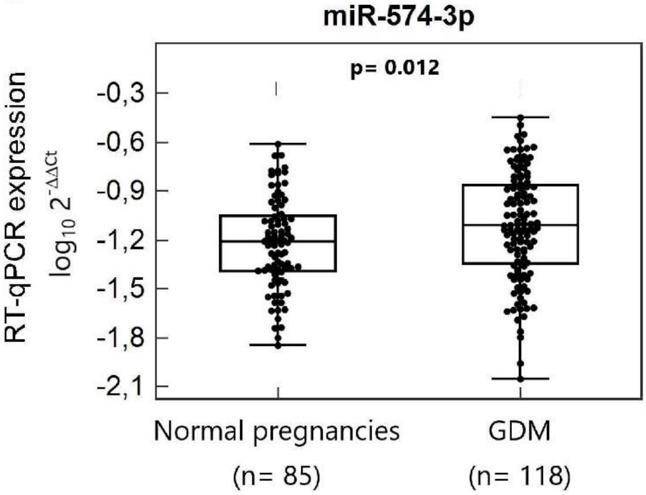

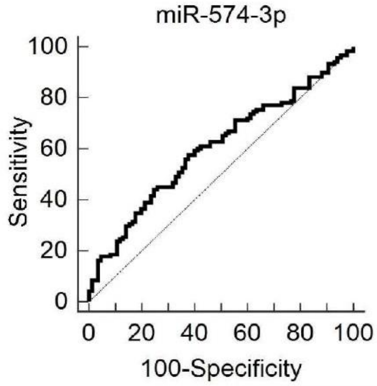

Area under the ROC curve (AUC)	0.603
Standard Error	0.0397
95% Confidence interval	0.532 to 0.671
Significance level P (Area=0.5)	0.0095

Estimated sensitivity at fixed specificity			
Specificity	Sensitivity	95% CI	Criterion
90.00	18.64	8.21 to 27.97	>0.158194774

Criterion	Sensitivity	95% CI	Specificity	95% CI	+LR	95% CI	-LR	95% CI
>0.070372444	57.63	48.2 - 66.7	62.35	51.2 - 72.6	1.53	1.1 - 2.1	0.68	0.5 - 0.9

T

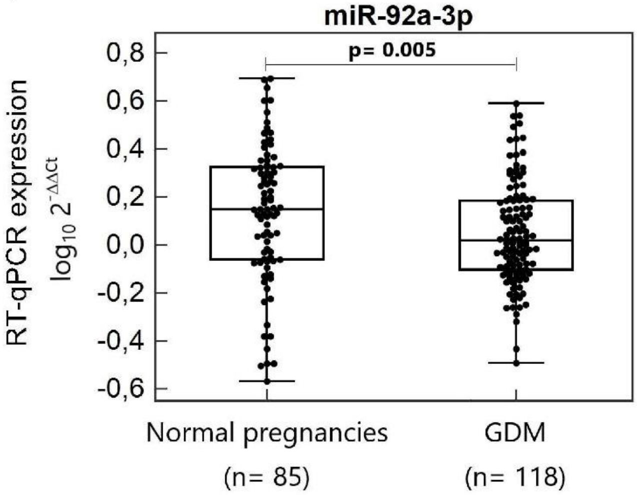

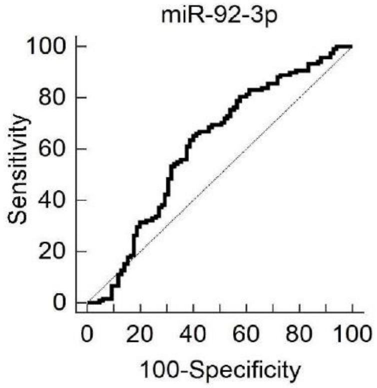

Area under the ROC curve (AUC)	0.614
Standard Error	0.0416
95% Confidence interval	0.543 to 0.681
Significance level P (Area=0.5)	0.0061

Estimated sensitivity at fixed specificity			
Specificity	Sensitivity	95% CI	Criterion
90.00	6.78	0.00 to 31.25	≤0.569554185

Criterion	Sensitivity	95% CI	Specificity	95% CI	+LR	95% CI	-LR	95% CI
≤1.301234196	65.25	55.9 - 73.8	60.00	48.8 - 70.5	1.63	1.2 - 2.2	0.58	0.4 - 0.8

U

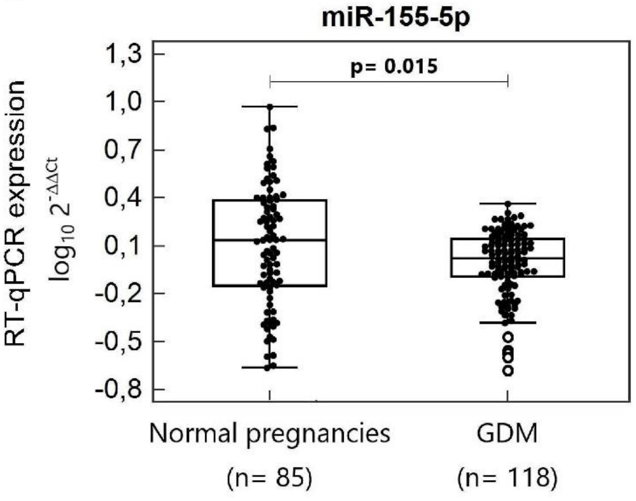

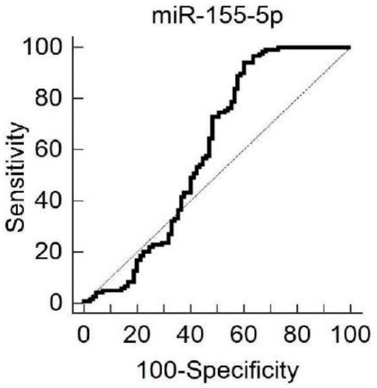

Area under the ROC curve (AUC)	0.600
Standard Error	0.0447
95% Confidence interval	0.529 to 0.668
Significance level P (Area=0.5)	0.0250

Estimated sensitivity at fixed specificity			
Specificity	Sensitivity	95% CI	Criterion
90.00	5.08	1.69 to 14.45	≤0.386873975

Criterion	Sensitivity	95% CI	Specificity	95% CI	+LR	95% CI	-LR	95% CI
≤1.72104999	94.07	88.2 - 97.6	40.00	29.5 - 51.2	1.57	1.3 - 1.9	0.15	0.07 - 0.3

V

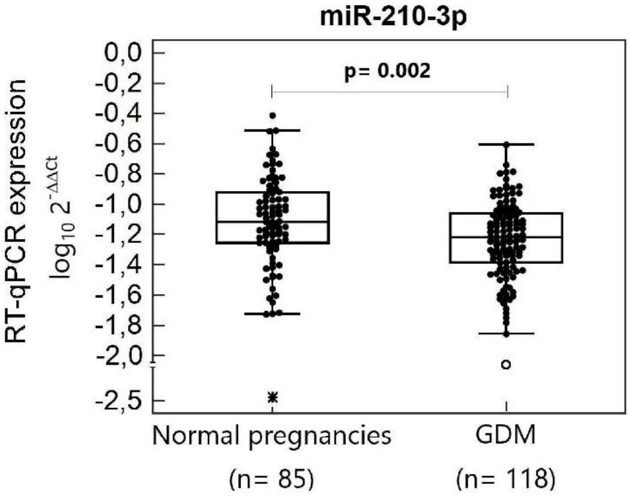

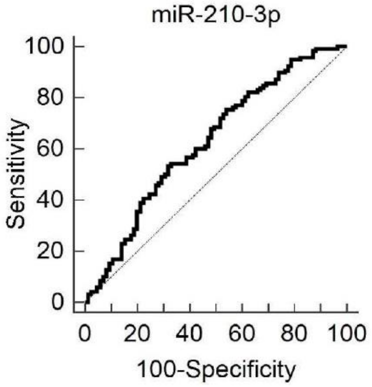

Area under the ROC curve (AUC)	0.627
Standard Error	0.0403
95% Confidence interval	0.557 to 0.694
Significance level P (Area=0.5)	0.0016

Estimated sensitivity at fixed specificity			
Specificity	Sensitivity	95% CI	Criterion
90.00	15.25	5.08 to 29.66	≤0.031493203

Criterion	Sensitivity	95% CI	Specificity	95% CI	+LR	95% CI	-LR	95% CI
≤0.0613105	53.39	44.0 - 62.6	68.24	57.2 - 77.9	1.68	1.2 - 2.4	0.68	0.5 - 0.9

Supplementary Figure S3: Aberrant microRNA expression profile in children descending from GDM complicated pregnancies irrespective of the clinical findings (overweight/obesity, prehypertension/hypertension, and/or valve problems and heart defects). (**A–S**) Up-regulation of miR-16-5p, miR-17-5p, miR-20a-5p, miR-20b-5p, miR-21-5p, miR-26a-5p, miR-29a-3p, miR-103a-3p, miR-125b-5p, miR-126-3p, miR-133a-3p, miR-143-3p, miR-146a-5p, miR-181a-5p, miR-195-5p, miR-199a-5p, miR-221-3p, miR-499a-5p, and miR-574-3p and (**T–V**) down-regulation of miR-92a-3p, miR-155-5p, and miR-210-3p was observed in children descending from GDM complicated pregnancies when the comparison to the controls irrespective of the clinical findings was performed. GDM, gestational diabetes mellitus.

A

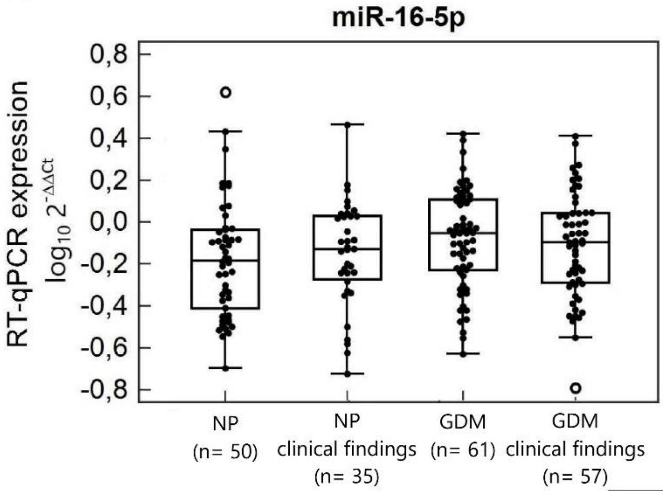

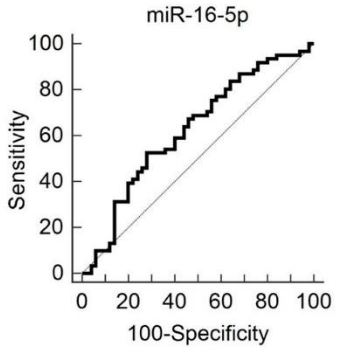

Area under the ROC curve (AUC)	0.624
Standard Error	0.0543
95% Confidence interval	0.527 to 0.714
Significance level P (Area=0.5)	0.0224

Estimated sensitivity at fixed specificity			
Specificity	Sensitivity	95% CI	Criterion
90.00	9.84	1.64 to 37.70	>1.496587503

Criterion	Sensitivity	95% CI	Specificity	95% CI	+LR	95% CI	-LR	95% CI
>0.846738909	52.46	39.3 - 65.4	72.00	57.5 - 83.8	1.87	1.1 - 3.1	0.66	0.5 - 0.9

B

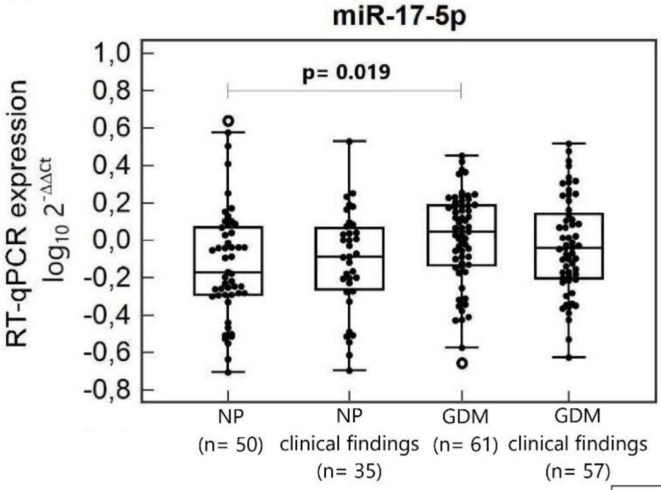

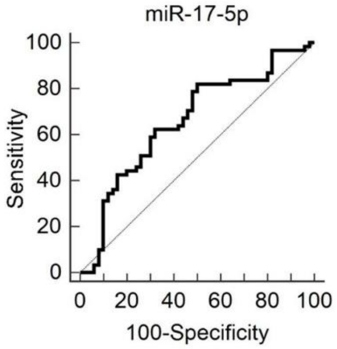

Area under the ROC curve (AUC)	0.658
Standard Error	0.0532
95% Confidence interval	0.562 to 0.746
Significance level P (Area=0.5)	0.0029

Estimated sensitivity at fixed specificity			
Specificity	Sensitivity	95% CI	Criterion
90.00	31.15	0.00 to 52.46	>1.476408484

Criterion	Sensitivity	95% CI	Specificity	95% CI	+LR	95% CI	-LR	95% CI
>0.658979863	81.97	70.0 - 90.6	50.00	35.5 - 64.5	1.64	1.2 - 2.2	0.36	0.2 - 0.7

C

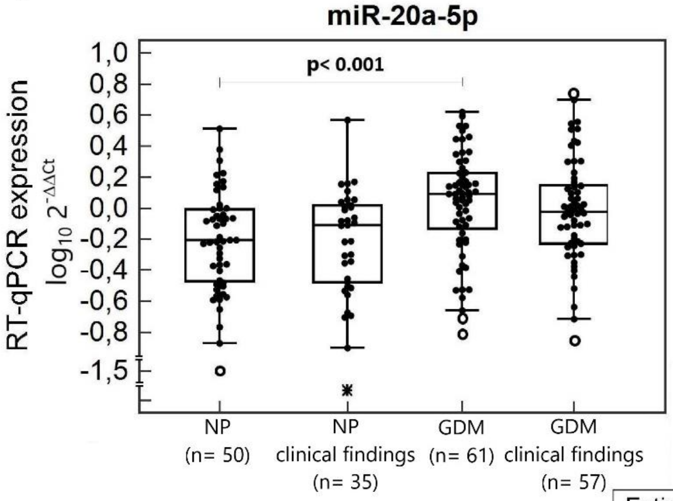

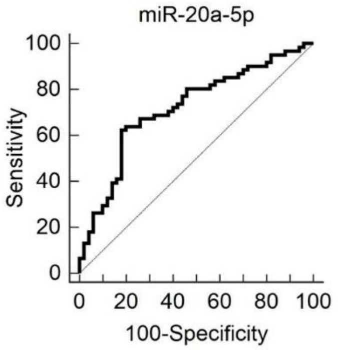

Area under the ROC curve (AUC)	0.719
Standard Error	0.0493
95% Confidence interval	0.626 to 0.800
Significance level P (Area=0.5)	<0.0001

Estimated sensitivity at fixed specificity			
Specificity	Sensitivity	95% CI	Criterion
90.00	29.51	11.48 to 60.66	>1.481534

Criterion	Sensitivity	95% CI	Specificity	95% CI	+LR	95% CI	-LR	95% CI
>1.042907	62.30	49.0 - 74.4	82.00	68.6 - 91.4	3.46	1.9 - 6.5	0.46	0.3 - 0.7

D

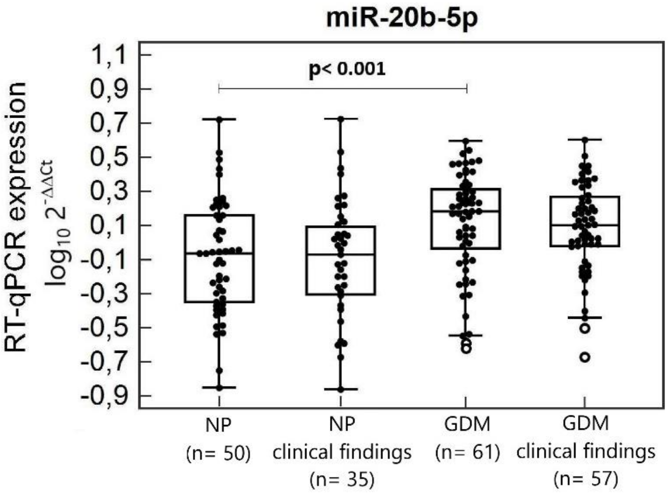

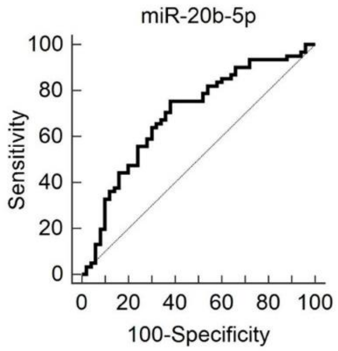

Area under the ROC curve (AUC)	0.696
Standard Error	0.0511
95% Confidence interval	0.602 to 0.780
Significance level P (Area=0.5)	0.0001

Estimated sensitivity at fixed specificity			
Specificity	Sensitivity	95% CI	Criterion
90.00	32.79	4.92 to 55.74	>1.813608

Criterion	Sensitivity	95% CI	Specificity	95% CI	+LR	95% CI	-LR	95% CI
>0.895453	75.41	62.7 - 85.5	62.00	47.2 - 75.3	1.98	1.4 - 2.9	0.40	0.2 - 0.6

E

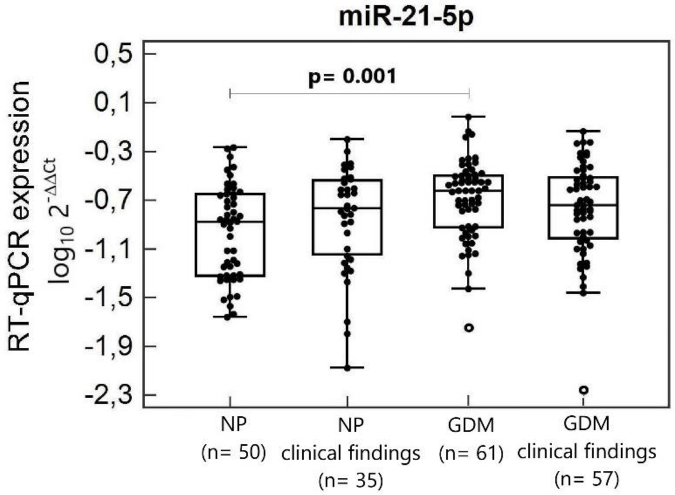

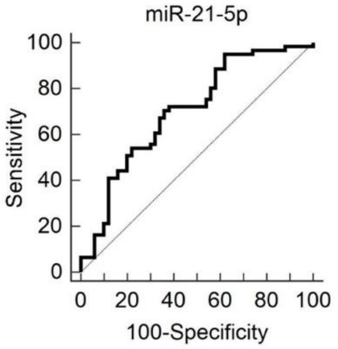

Area under the ROC curve (AUC)	0.703
Standard Error	0.0505
95% Confidence interval	0.608 to 0.786
Significance level P (Area=0.5)	0.0001

Estimated sensitivity at fixed specificity			
Specificity	Sensitivity	95% CI	Criterion
90.00	21.31	4.92 to 50.82	>0.323192755

Criterion	Sensitivity	95% CI	Specificity	95% CI	+LR	95% CI	-LR	95% CI
>0.160926828	70.49	57.4 - 81.5	64.00	49.2 - 77.1	1.96	1.3 - 2.9	0.46	0.3 - 0.7

F

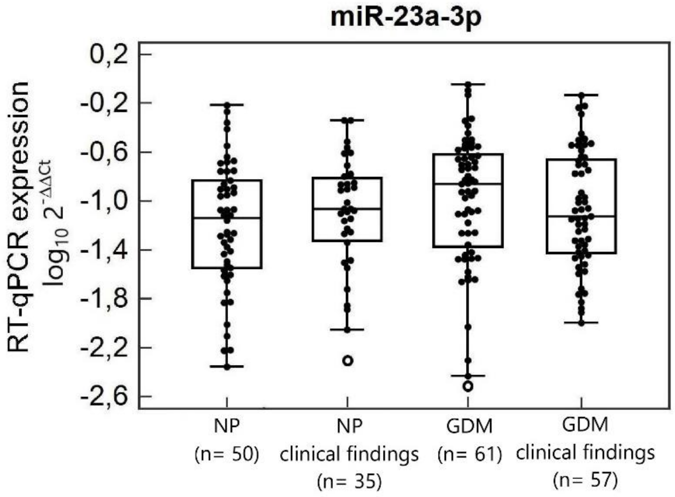

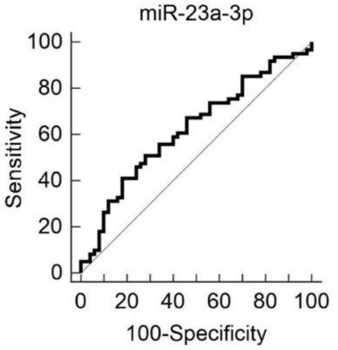

Area under the ROC curve (AUC)	0.622
Standard Error	0.0535
95% Confidence interval	0.525 to 0.712
Significance level P (Area=0.5)	0.0231

Estimated sensitivity at fixed specificity			
Specificity	Sensitivity	95% CI	Criterion
90.00	26.23	6.56 to 47.54	>0.229132348

Criterion	Sensitivity	95% CI	Specificity	95% CI	+LR	95% CI	-LR	95% CI
>0.176739191	40.98	28.6 - 54.3	82.00	68.6 - 91.4	2.28	1.2 - 4.4	0.72	0.6 - 0.9

G

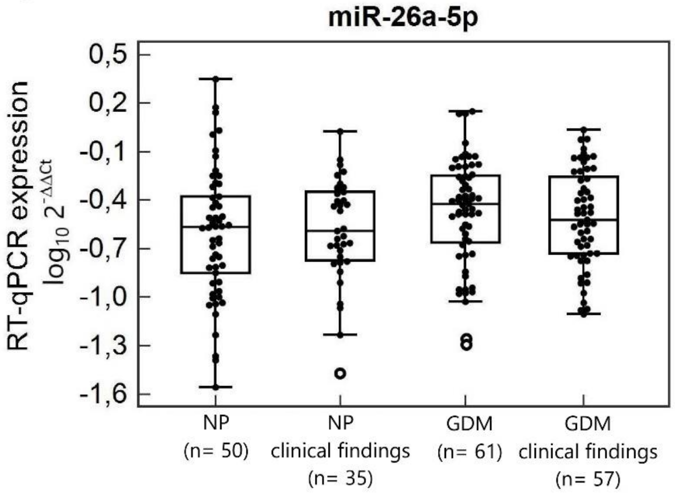

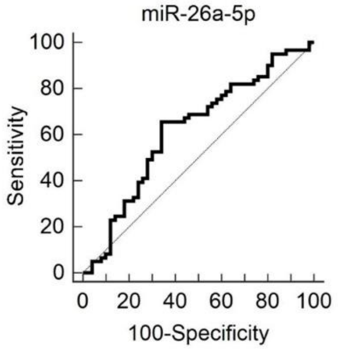

Area under the ROC curve (AUC)	0.619
Standard Error	0.0547
95% Confidence interval	0.522 to 0.710
Significance level P (Area=0.5)	0.0292

Estimated sensitivity at fixed specificity			
Specificity	Sensitivity	95% CI	Criterion
90.00	8.20	0.00 to 32.79	>0.746042247

Criterion	Sensitivity	95% CI	Specificity	95% CI	+LR	95% CI	-LR	95% CI
>0.314884334	65.57	52.3 - 77.3	66.00	51.2 - 78.8	1.93	1.3 - 3.0	0.52	0.3 - 0.8

H

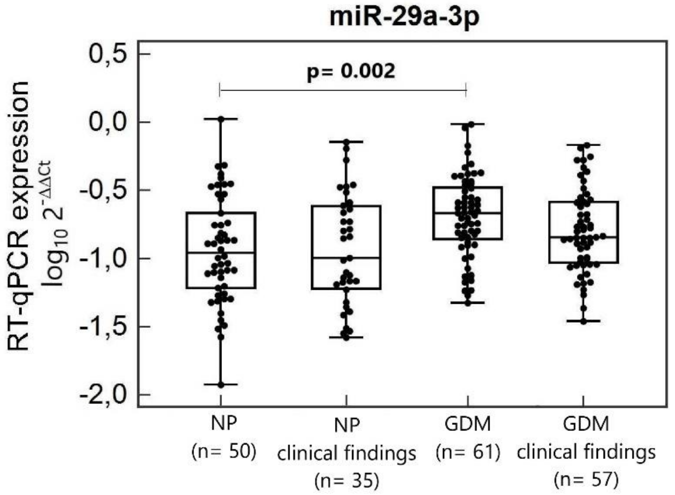

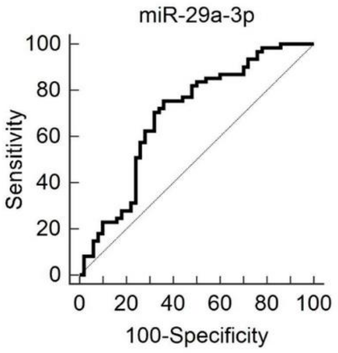

Area under the ROC curve (AUC)	0.693
Standard Error	0.0522
95% Confidence interval	0.599 to 0.777
Significance level P (Area=0.5)	0.0002

Estimated sensitivity at fixed specificity			
Specificity	Sensitivity	95% CI	Criterion
90.00	22.95	9.84 to 55.74	>0.351278964

Criterion	Sensitivity	95% CI	Specificity	95% CI	+LR	95% CI	-LR	95% CI
>0.139968015	75.41	62.7 - 85.5	64.00	49.2 - 77.1	2.09	1.4 - 3.1	0.38	0.2 - 0.6

I

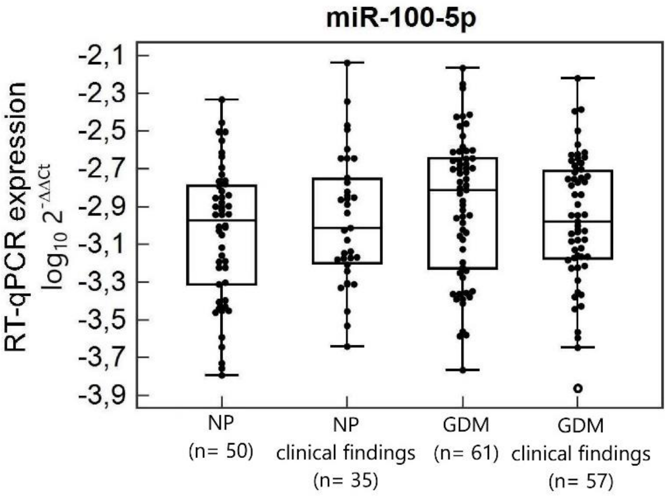

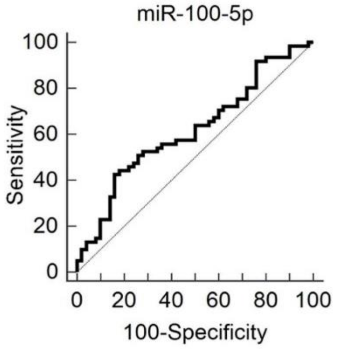

Area under the ROC curve (AUC)	0.617
Standard Error	0.0537
95% Confidence interval	0.520 to 0.708
Significance level P (Area=0.5)	0.0292

Estimated sensitivity at fixed specificity			
Specificity	Sensitivity	95% CI	Criterion
90.00	22.95	8.20 to 52.46	>0.002435069

Criterion	Sensitivity	95% CI	Specificity	95% CI	+LR	95% CI	-LR	95% CI
>0.001860482	42.62	30.0 - 55.9	84.00	70.9 - 92.8	2.66	1.3 - 5.4	0.68	0.5 - 0.9

J

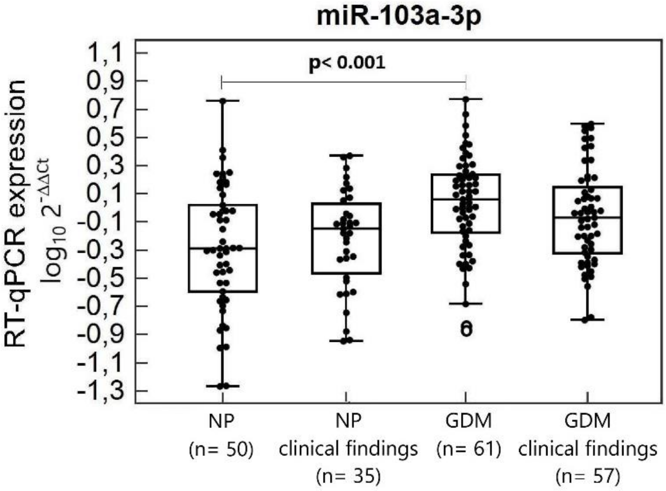

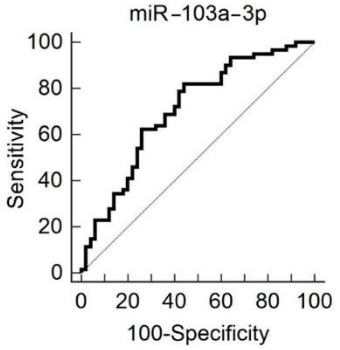

Area under the ROC curve (AUC)	0.708
Standard Error	0.0502
95% Confidence interval	0.614 to 0.791
Significance level P (Area=0.5)	<0.0001

Estimated sensitivity at fixed specificity			
Specificity	Sensitivity	95% CI	Criterion
90.00	22.95	8.20 to 38.83	>1.768871485

Criterion	Sensitivity	95% CI	Specificity	95% CI	+LR	95% CI	-LR	95% CI
>0.524775066	81.97	70.0 - 90.6	56.00	41.3 - 70.0	1.86	1.3 - 2.6	0.32	0.2 - 0.6

K

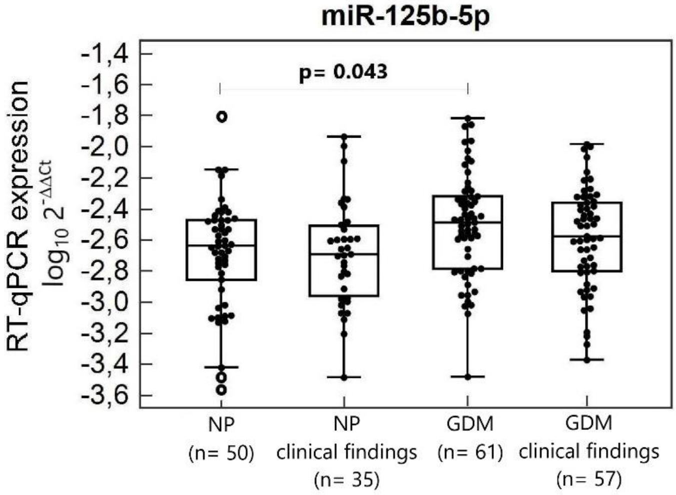

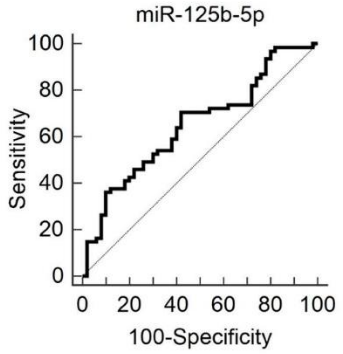

Area under the ROC curve (AUC)	0.649
Standard Error	0.0524
95% Confidence interval	0.552 to 0.737
Significance level P (Area=0.5)	0.0045

Estimated sensitivity at fixed specificity			
Specificity	Sensitivity	95% CI	Criterion
90.00	36.07	14.75 to 54.10	>0.004081449

Criterion	Sensitivity	95% CI	Specificity	95% CI	+LR	95% CI	-LR	95% CI
>0.002504764	70.49	57.4 - 81.5	58.00	43.2 - 71.8	1.68	1.2 - 2.4	0.51	0.3 - 0.8

L

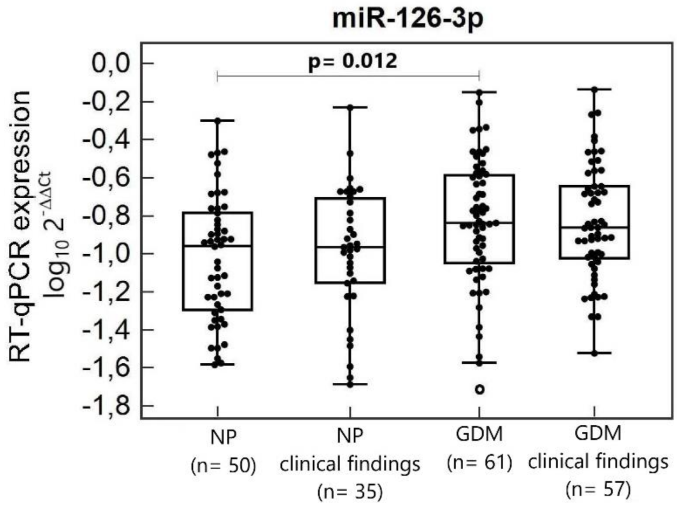

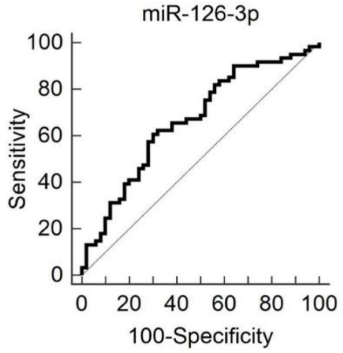

Area under the ROC curve (AUC)	0.667
Standard Error	0.0520
95% Confidence interval	0.571 to 0.753
Significance level P (Area=0.5)	0.0014

Estimated sensitivity at fixed specificity			
Specificity	Sensitivity	95% CI	Criterion
90.00	24.59	8.20 to 44.26	>0.260164721

Criterion	Sensitivity	95% CI	Specificity	95% CI	+LR	95% CI	-LR	95% CI
>0.133541075	60.66	47.3 - 72.9	70.00	55.4 - 82.1	2.02	1.3 - 3.2	0.56	0.4 - 0.8

M

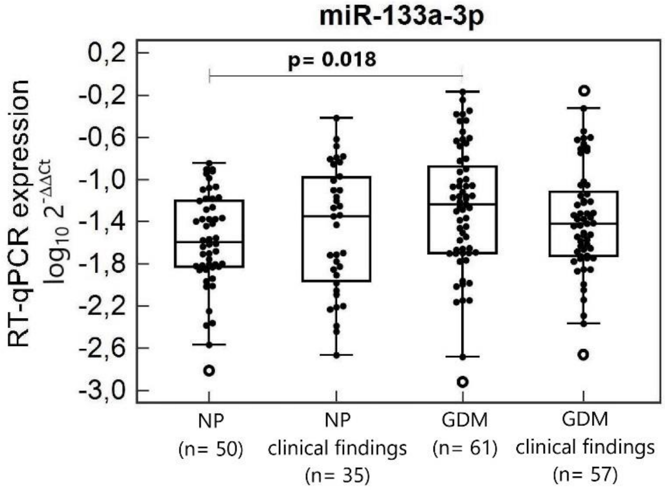

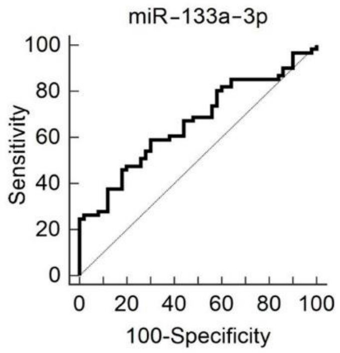

Area under the ROC curve (AUC)	0.663
Standard Error	0.0513
95% Confidence interval	0.567 to 0.750
Significance level P (Area=0.5)	0.0015

Estimated sensitivity at fixed specificity			
Specificity	Sensitivity	95% CI	Criterion
90.00	27.87	13.11 to 42.62	>0.102754776

Criterion	Sensitivity	95% CI	Specificity	95% CI	+LR	95% CI	-LR	95% CI
>0.042944859	59.02	45.7 - 71.4	70.00	55.4 - 82.1	1.97	1.2 - 3.2	0.59	0.4 - 0.8

N

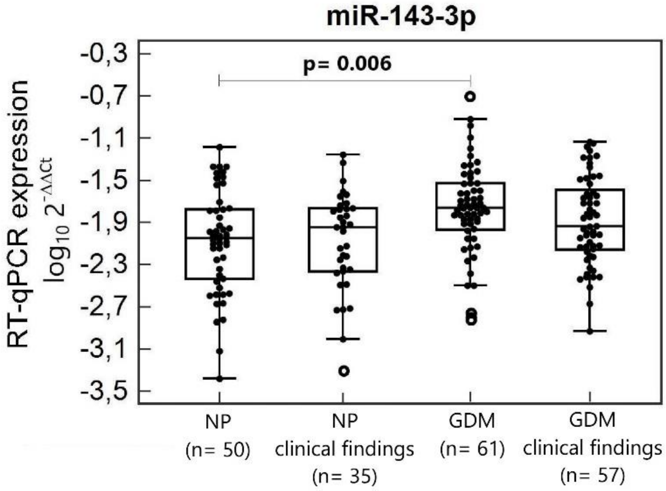

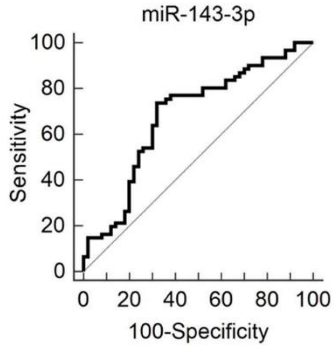

Area under the ROC curve (AUC)	0.681
Standard Error	0.0524
95% Confidence interval	0.586 to 0.766
Significance level P (Area=0.5)	0.0006

Estimated sensitivity at fixed specificity			
Specificity	Sensitivity	95% CI	Criterion
90.00	16.39	6.56 to 31.15	>0.036813583

Criterion	Sensitivity	95% CI	Specificity	95% CI	+LR	95% CI	-LR	95% CI
>0.011820045	73.77	60.9 - 84.2	68.00	53.3 - 80.5	2.31	1.5 - 3.5	0.39	0.2 - 0.6

O

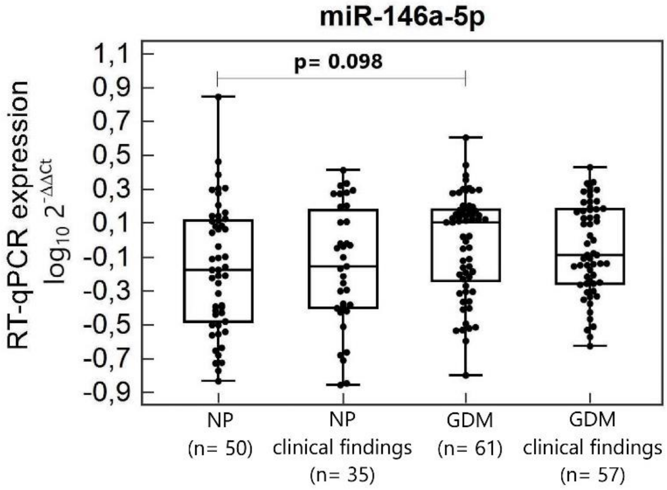

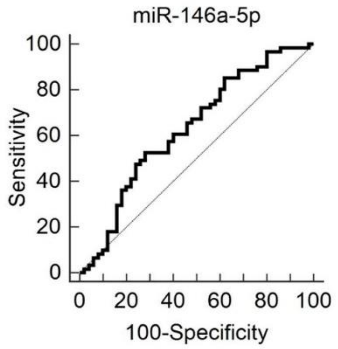

Area under the ROC curve (AUC)	0.630
Standard Error	0.0542
95% Confidence interval	0.534 to 0.720
Significance level P (Area=0.5)	0.0161

Estimated sensitivity at fixed specificity			
Specificity	Sensitivity	95% CI	Criterion
90.00	9.84	1.64 to 36.07	>1.978207675

Criterion	Sensitivity	95% CI	Specificity	95% CI	+LR	95% CI	-LR	95% CI
>1.208562374	52.46	39.3 - 65.4	72.00	57.5 - 83.8	1.87	1.1 - 3.1	0.66	0.5 - 0.9

P

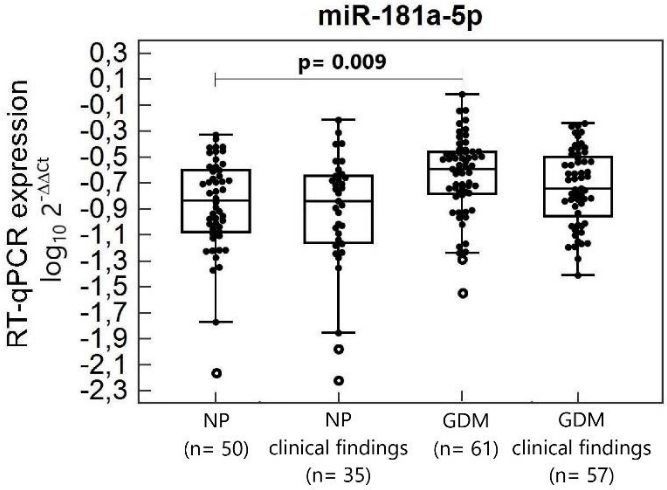

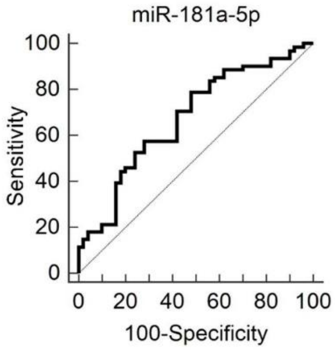

Area under the ROC curve (AUC)	0.674
Standard Error	0.0517
95% Confidence interval	0.578 to 0.760
Significance level P (Area=0.5)	0.0008

Estimated sensitivity at fixed specificity			
Specificity	Sensitivity	95% CI	Criterion
90.00	21.31	11.48 to 50.82	>0.354784047

Criterion	Sensitivity	95% CI	Specificity	95% CI	+LR	95% CI	-LR	95% CI
>0.153329532	78.69	66.3 - 88.1	52.00	37.4 - 66.3	1.64	1.2 - 2.3	0.41	0.2 - 0.7

Q

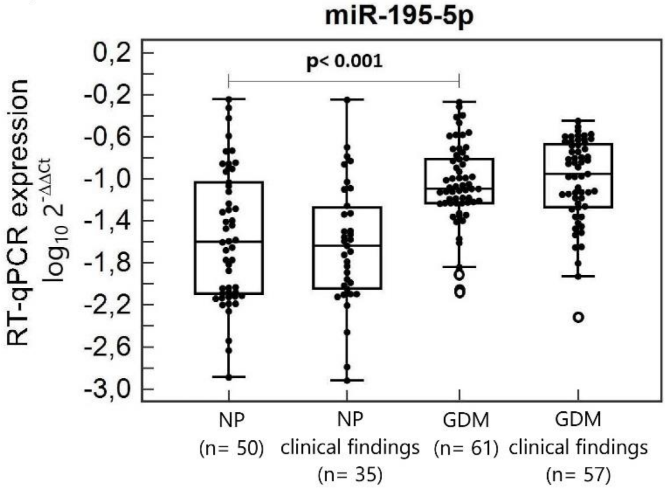

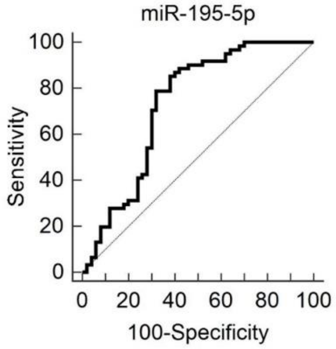

Area under the ROC curve (AUC)	0.730
Standard Error	0.0516
95% Confidence interval	0.637 to 0.809
Significance level P (Area=0.5)	<0.0001

Estimated sensitivity at fixed specificity			
Specificity	Sensitivity	95% CI	Criterion
90.00	19.67	4.92 to 36.07	>0.183009328

Criterion	Sensitivity	95% CI	Specificity	95% CI	+LR	95% CI	-LR	95% CI
>0.039818758	85.25	73.8 - 93.0	62.00	47.2 - 75.3	2.24	1.6 - 3.2	0.24	0.1 - 0.5

R

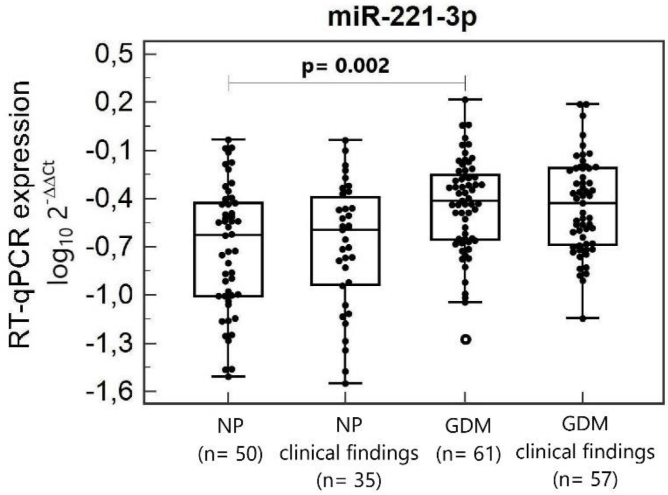

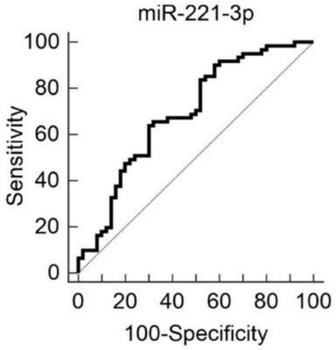

Area under the ROC curve (AUC)	0.692
Standard Error	0.0513
95% Confidence interval	0.598 to 0.777
Significance level P (Area=0.5)	0.0002

Estimated sensitivity at fixed specificity			
Specificity	Sensitivity	95% CI	Criterion
90.00	18.03	4.92 to 47.54	>0.650022942

Criterion	Sensitivity	95% CI	Specificity	95% CI	+LR	95% CI	-LR	95% CI
>0.322303976	63.93	50.6 - 75.8	70.00	55.4 - 82.1	2.13	1.3 - 3.4	0.52	0.4 - 0.8

S

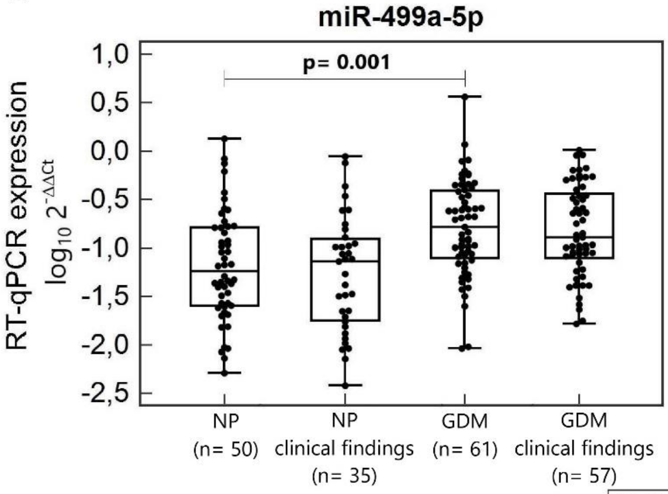

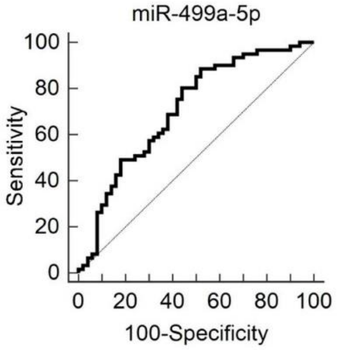

Area under the ROC curve (AUC)	0.709
Standard Error	0.0505
95% Confidence interval	0.615 to 0.791
Significance level P (Area=0.5)	<0.0001

Estimated sensitivity at fixed specificity			
Specificity	Sensitivity	95% CI	Criterion
90.00	29.51	3.28 to 50.82	>0.32173153

Criterion	Sensitivity	95% CI	Specificity	95% CI	+LR	95% CI	-LR	95% CI
>0.04701734	88.52	77.8 - 95.3	48.00	33.7 - 62.6	1.70	1.3 - 2.3	0.24	0.1 - 0.5

T

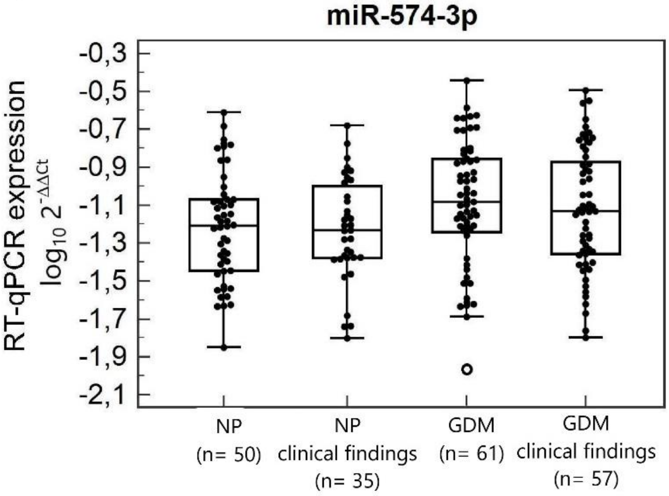

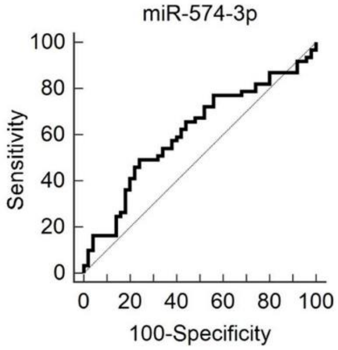

Area under the ROC curve (AUC)	0.610
Standard Error	0.0541
95% Confidence interval	0.513 to 0.702
Significance level P (Area=0.5)	0.0413

Estimated sensitivity at fixed specificity			
Specificity	Sensitivity	95% CI	Criterion
90.00	16.39	6.56 to 36.07	>0.1622597

Criterion	Sensitivity	95% CI	Specificity	95% CI	+LR	95% CI	-LR	95% CI
>0.085096123	49.18	36.1 - 62.3	76.00	61.8 - 86.9	2.05	1.2 - 3.6	0.67	0.5 - 0.9

U

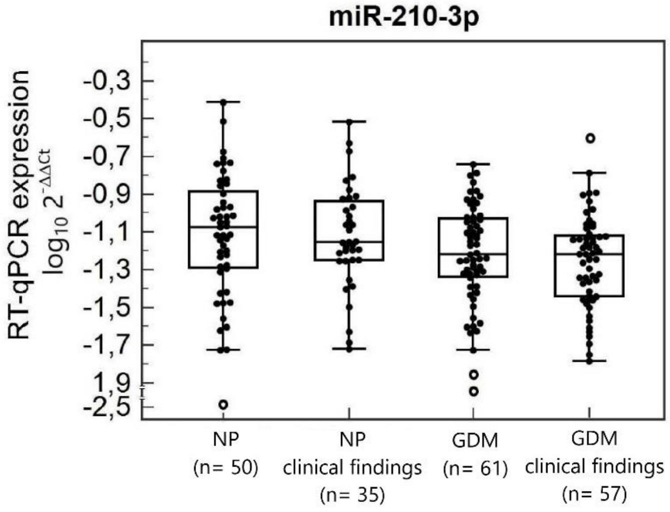

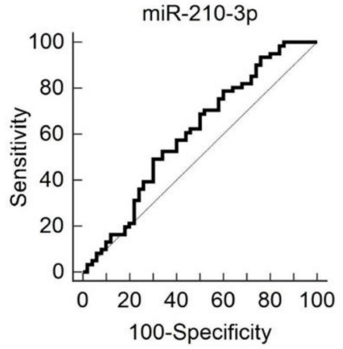

Area under the ROC curve (AUC)	0.604
Standard Error	0.0552
95% Confidence interval	0.507 to 0.696
Significance level P (Area=0.5)	0.0595

Estimated sensitivity at fixed specificity			
Specificity	Sensitivity	95% CI	Criterion
90.00	13.11	3.28 to 31.15	≤0.02602497

Criterion	Sensitivity	95% CI	Specificity	95% CI	+LR	95% CI	-LR	95% CI
≤0.05774473	49.18	36.1 - 62.3	70.00	55.4 - 82.1	1.64	1.0 - 2.7	0.73	0.5 - 1.0

Supplementary Figure S4: Aberrant microRNA expression profile in children descending from GDM complicated pregnancies with normal clinical findings. (**A–T**) Up-regulation of miR-16-5p, miR-17-5p, miR-20a-5p, miR-20b-5p, miR-21-5p, miR-23a-3p, miR-26a-5p, miR-29a-3p, miR-100-5p, miR-103a-3p, miR-125b-5p, miR-126-3p, miR-133a-3p, miR-143-3p, miR-146a-5p, miR-181a-5p, miR-195-5p, miR-221-3p, miR-499a-5p, and miR-574-3p and (**U**) down-regulation of miR-210-3p was observed in children descending from GDM complicated pregnancies with normal clinical findings, when the comparison to the controls with normal clinical findings was performed. NP, normal pregnancies; GDM, gestational diabetes mellitus.

A

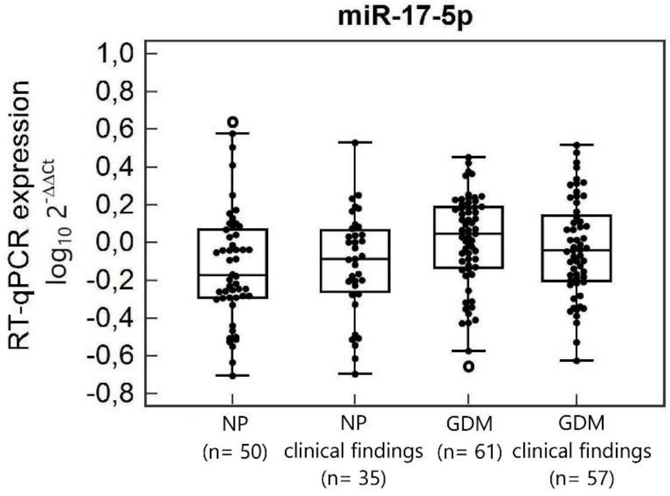

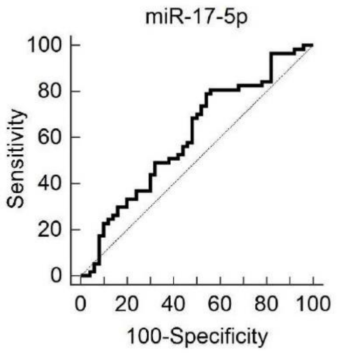

Area under the ROC curve (AUC)	0.606
Standard Error	0.0555
95% Confidence interval	0.507 to 0.699
Significance level P (Area=0.5)	0.0561

Estimated sensitivity at fixed specificity			
Specificity	Sensitivity	95% CI	Criterion
90.00	22.81	1.75 to 40.35	>1.476408484

Criterion	Sensitivity	95% CI	Specificity	95% CI	+LR	95% CI	-LR	95% CI
>0.599240692	78.95	66.1 - 88.6	46.00	31.8 - 60.7	1.46	1.1 - 2.0	0.46	0.3 - 0.8

B

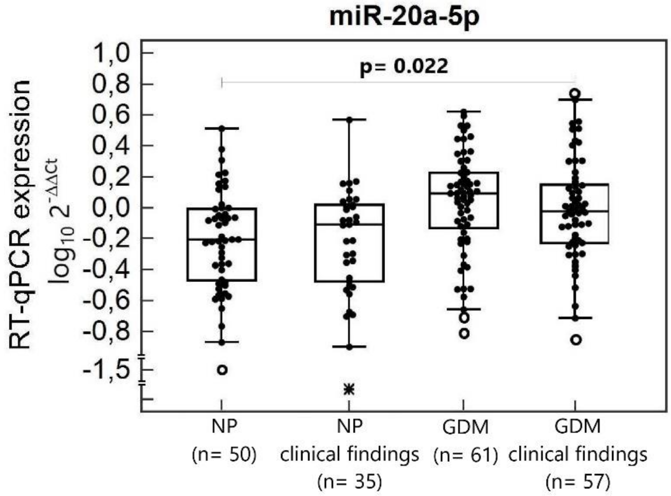

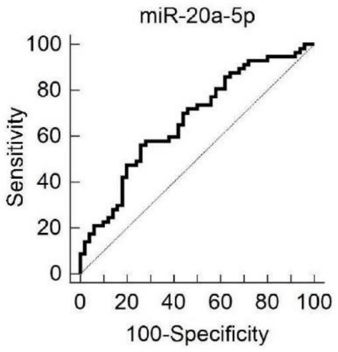

Area under the ROC curve (AUC)	0.671
Standard Error	0.0523
95% Confidence interval	0.573 to 0.759
Significance level P (Area=0.5)	0.0011

Estimated sensitivity at fixed specificity			
Specificity	Sensitivity	95% CI	Criterion
90.00	22.81	10.53 to 43.86	>1.481534

Criterion	Sensitivity	95% CI	Specificity	95% CI	+LR	95% CI	-LR	95% CI
>0.901802	56.14	42.4 - 69.3	74.00	59.7 - 85.4	2.16	1.3 - 3.6	0.59	0.4 - 0.8

C

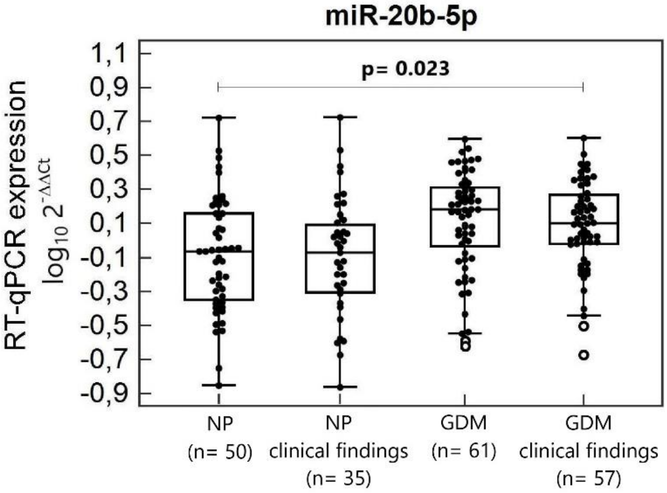

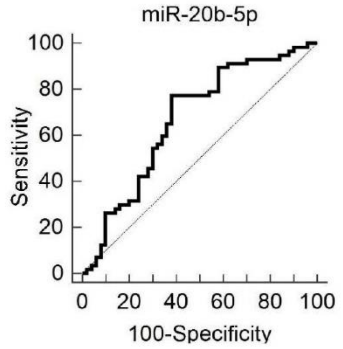

Area under the ROC curve (AUC)	0.669
Standard Error	0.0540
95% Confidence interval	0.571 to 0.757
Significance level P (Area=0.5)	0.0018

Estimated sensitivity at fixed specificity			
Specificity	Sensitivity	95% CI	Criterion
90.00	26.32	5.26 to 45.61	>1.813608

Criterion	Sensitivity	95% CI	Specificity	95% CI	+LR	95% CI	-LR	95% CI
>0.895453	77.19	64.2 - 87.3	62.00	47.2 - 75.3	2.03	1.4 - 3.0	0.37	0.2 - 0.6

D

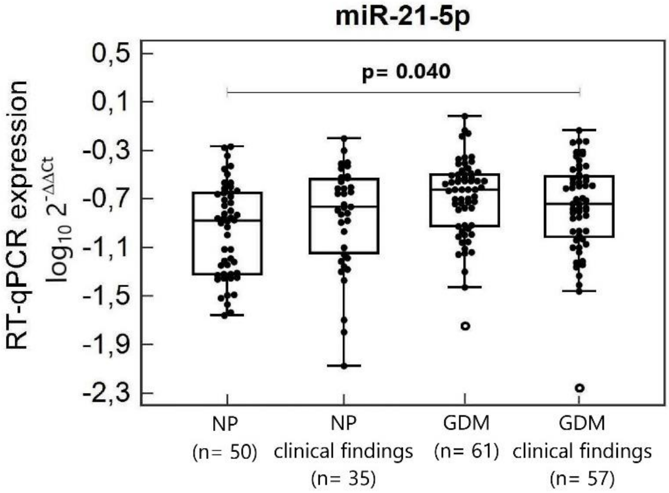

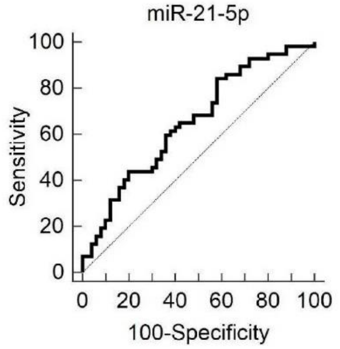

Area under the ROC curve (AUC)	0.653
Standard Error	0.0532
95% Confidence interval	0.554 to 0.742
Significance level P (Area=0.5)	0.0041

Estimated sensitivity at fixed specificity			
Specificity	Sensitivity	95% CI	Criterion
90.00	22.81	7.02 to 42.11	>0.323192755

Criterion	Sensitivity	95% CI	Specificity	95% CI	+LR	95% CI	-LR	95% CI
>0.077419504	84.21	72.1 - 92.5	42.00	28.2 - 56.8	1.45	1.1 - 1.9	0.38	0.2 - 0.7

E

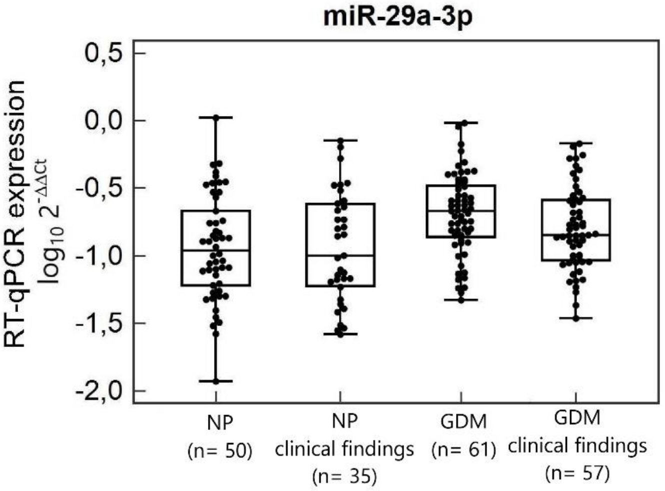

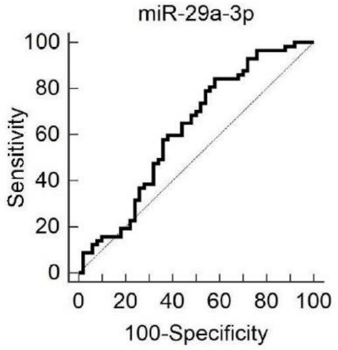

Area under the ROC curve (AUC)	0.621
Standard Error	0.0557
95% Confidence interval	0.522 to 0.713
Significance level P (Area=0.5)	0.0297

Estimated sensitivity at fixed specificity			
Specificity	Sensitivity	95% CI	Criterion
90.00	15.79	7.02 to 33.33	>0.351278964

Criterion	Sensitivity	95% CI	Specificity	95% CI	+LR	95% CI	-LR	95% CI
>0.086833444	84.21	72.1 - 92.5	42.00	28.2 - 56.8	1.45	1.1 - 1.9	0.38	0.2 - 0.7

F

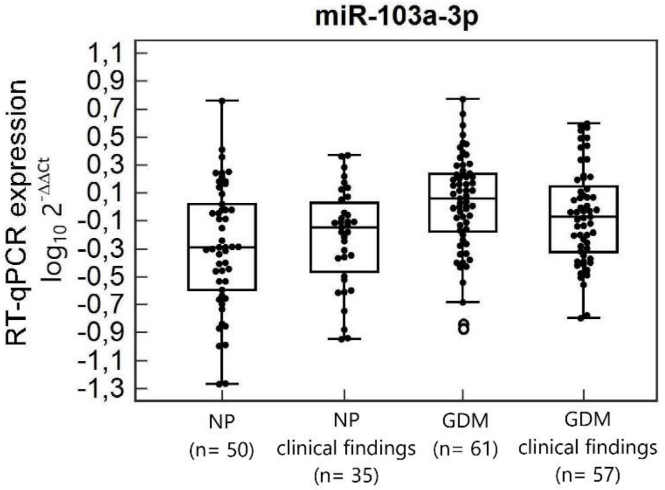

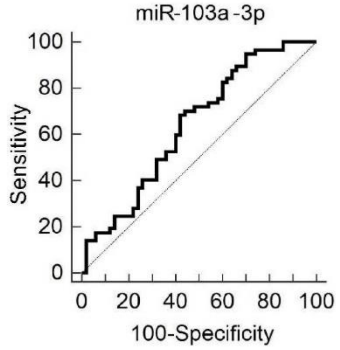

Area under the ROC curve (AUC)	0.639
Standard Error	0.0544
95% Confidence interval	0.541 to 0.730
Significance level P (Area=0.5)	0.0105

Estimated sensitivity at fixed specificity			
Specificity	Sensitivity	95% CI	Criterion
90.00	17.54	8.77 to 29.82	>1.768871485

Criterion	Sensitivity	95% CI	Specificity	95% CI	+LR	95% CI	-LR	95% CI
>0.580303016	68.42	54.8 - 80.1	58.00	43.2 - 71.8	1.63	1.1 - 2.4	0.54	0.3 - 0.9

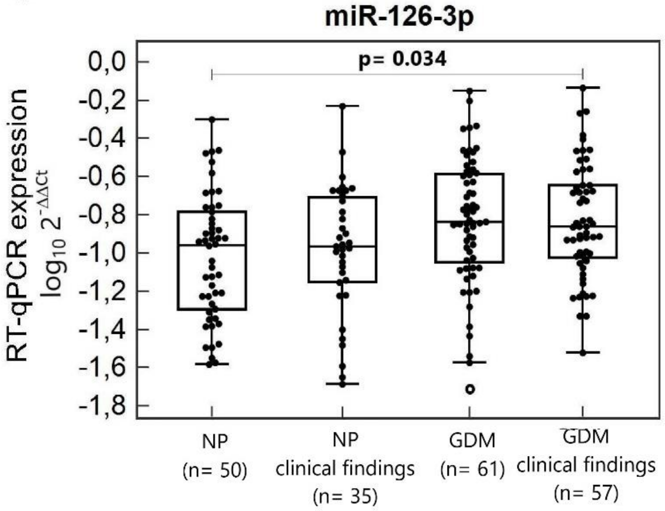

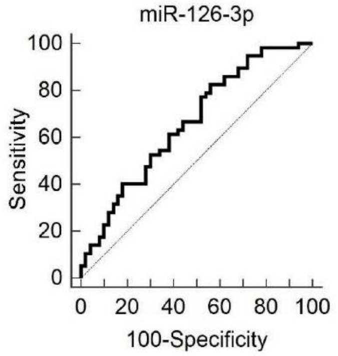

Area under the ROC curve (AUC)	0.661
Standard Error	0.0529
95% Confidence interval	0.563 to 0.750
Significance level P (Area=0.5)	0.0023

Estimated sensitivity at fixed specificity			
Specificity	Sensitivity	95% CI	Criterion
90.00	22.81	8.77 to 43.86	>0.260164721

Criterion	Sensitivity	95% CI	Specificity	95% CI	+LR	95% CI	-LR	95% CI
>0.076398154	82.46	70.1 - 91.3	44.00	30.0 - 58.7	1.47	1.1 - 1.9	0.40	0.2 - 0.8

H

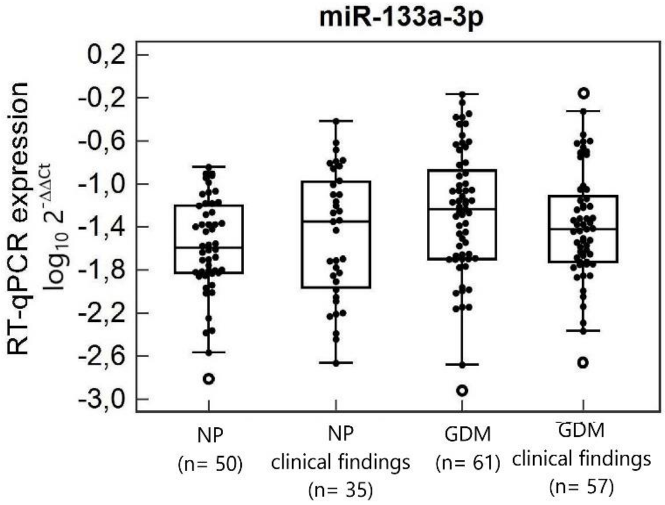

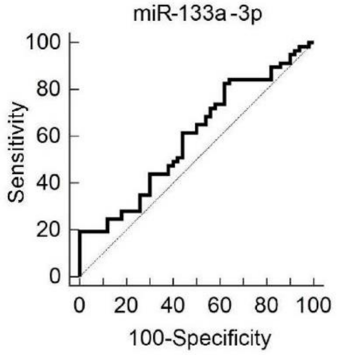

Area under the ROC curve (AUC)	0.597
Standard Error	0.0552
95% Confidence interval	0.498 to 0.691
Significance level P (Area=0.5)	0.0780

Estimated sensitivity at fixed specificity			
Specificity	Sensitivity	95% CI	Criterion
90.00	19.30	8.77 to 29.82	>0.102754776

Criterion	Sensitivity	95% CI	Specificity	95% CI	+LR	95% CI	-LR	95% CI
>0.01753083	82.46	70.1 - 91.3	38.00	24.7 - 52.8	1.33	1.0 - 1.7	0.46	0.2 - 0.9

I

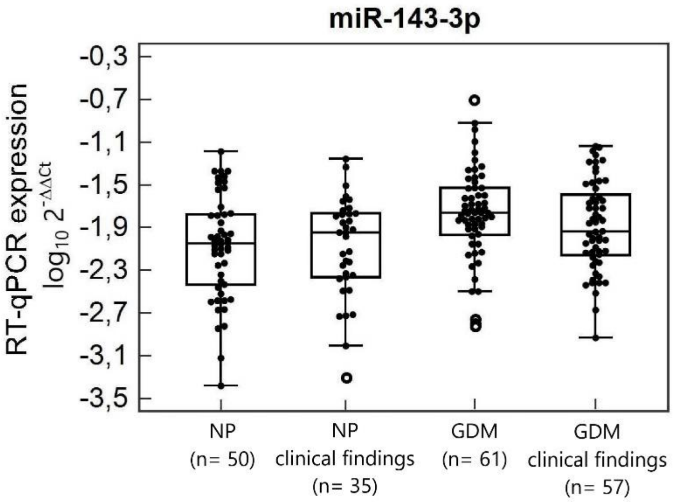

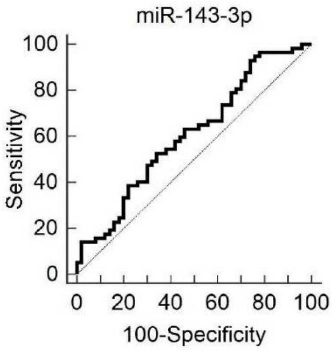

Area under the ROC curve (AUC)	0.608
Standard Error	0.0550
95% Confidence interval	0.509 to 0.701
Significance level P (Area=0.5)	0.0493

Estimated sensitivity at fixed specificity			
Specificity	Sensitivity	95% CI	Criterion
90.00	15.79	7.02 to 31.35	>0.036813583

Criterion	Sensitivity	95% CI	Specificity	95% CI	+LR	95% CI	-LR	95% CI
>0.003670539	92.98	83.0 - 98.1	26.00	14.6 - 40.3	1.26	1.1 - 1.5	0.27	0.09 - 0.8

J

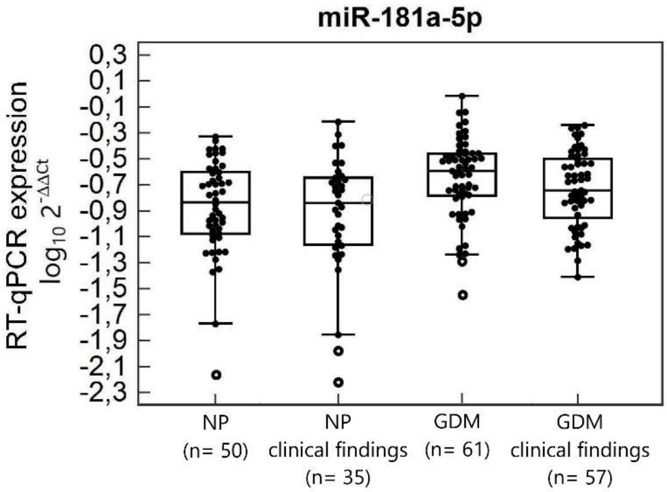

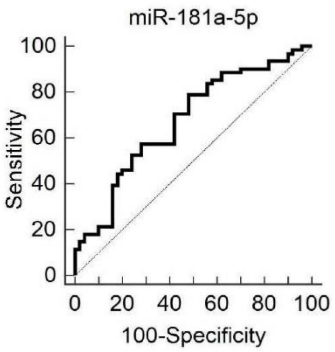

Area under the ROC curve (AUC)	0.603
Standard Error	0.0550
95% Confidence interval	0.504 to 0.696
Significance level P (Area=0.5)	0.0608

Estimated sensitivity at fixed specificity			
Specificity	Sensitivity	95% CI	Criterion
90.00	19.30	8.77 to 37.02	>0.354784047

Criterion	Sensitivity	95% CI	Specificity	95% CI	+LR	95% CI	-LR	95% CI
>0.131424946	73.68	60.3 - 84.5	48.00	33.7 - 62.6	1.42	1.0 - 1.9	0.55	0.3 - 0.9

K

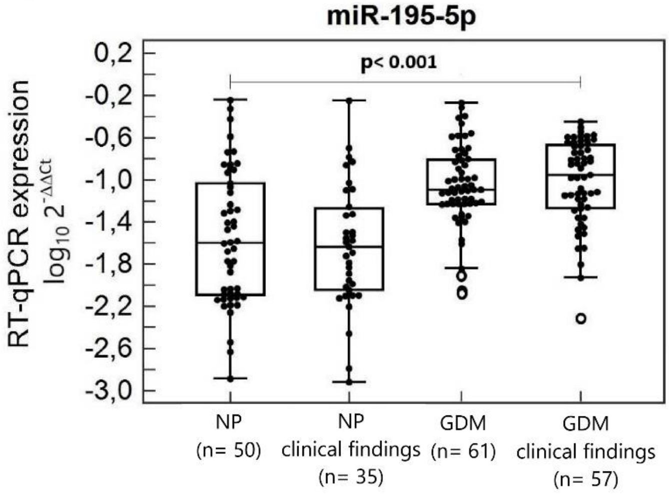

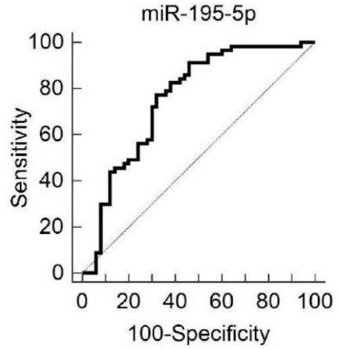

Area under the ROC curve (AUC)	0.755
Standard Error	0.0491
95% Confidence interval	0.662 to 0.833
Significance level P (Area=0.5)	<0.0001

Estimated sensitivity at fixed specificity			
Specificity	Sensitivity	95% CI	Criterion
90.00	29.82	0.00 to 50.88	>0.183009328

Criterion	Sensitivity	95% CI	Specificity	95% CI	+LR	95% CI	-LR	95% CI
>0.026151807	91.23	80.7 - 97.1	54.00	39.3 - 68.2	1.98	1.5 - 2.7	0.16	0.07 - 0.4

L

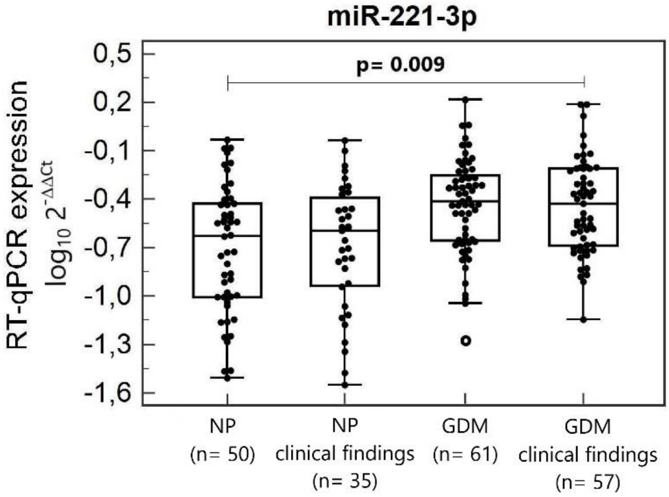

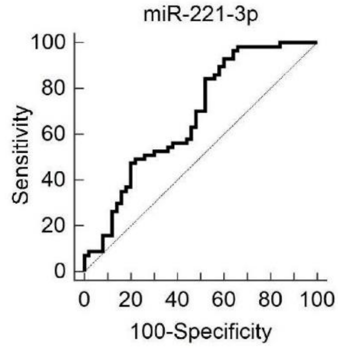

Area under the ROC curve (AUC)	0.679
Standard Error	0.0528
95% Confidence interval	0.581 to 0.766
Significance level P (Area=0.5)	0.0007

Estimated sensitivity at fixed specificity			
Specificity	Sensitivity	95% CI	Criterion
90.00	15.79	3.51 to 40.35	>0.650022942

Criterion	Sensitivity	95% CI	Specificity	95% CI	+LR	95% CI	-LR	95% CI
>0.137483943	92.98	83.0 - 98.1	40.00	26.4 - 54.8	1.55	1.2 - 2.0	0.18	0.06 - 0.5

M

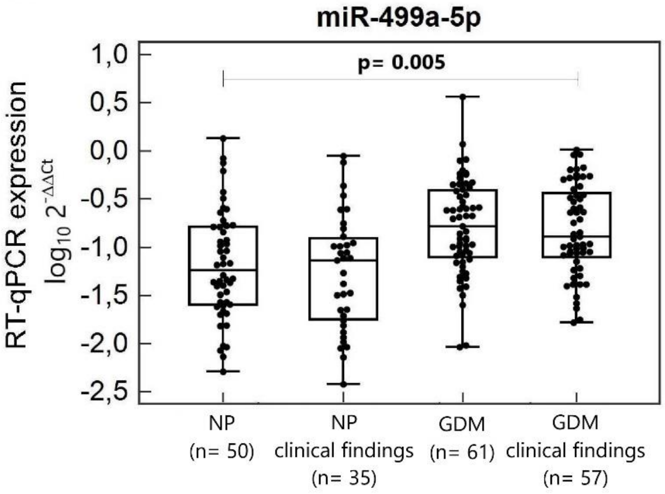

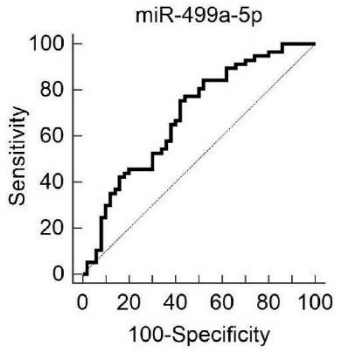

Area under the ROC curve (AUC)	0.688
Standard Error	0.0520
95% Confidence interval	0.591 to 0.774
Significance level P (Area=0.5)	0.0003

Estimated sensitivity at fixed specificity			
Specificity	Sensitivity	95% CI	Criterion
90.00	29.82	3.51 to 49.12	>0.32173153

Criterion	Sensitivity	95% CI	Specificity	95% CI	+LR	95% CI	-LR	95% CI
>0.077646272	75.44	62.2 - 85.9	58.00	43.2 - 71.8	1.80	1.3 - 2.6	0.42	0.3 - 0.7

N

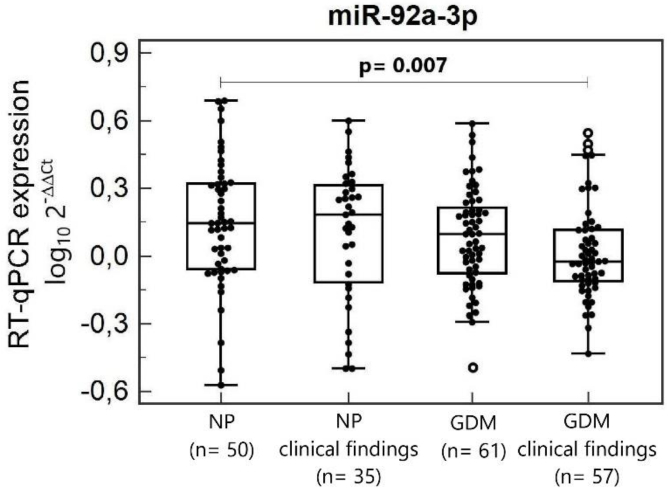

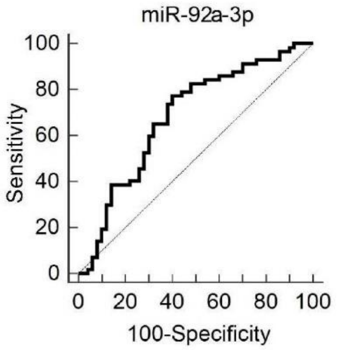

Area under the ROC curve (AUC)	0.682
Standard Error	0.0532
95% Confidence interval	0.585 to 0.768
Significance level P (Area=0.5)	0.0006

Estimated sensitivity at fixed specificity			
Specificity	Sensitivity	95% CI	Criterion
90.00	19.30	0.00 to 45.61	≤0.720377192

Criterion	Sensitivity	95% CI	Specificity	95% CI	+LR	95% CI	-LR	95% CI
≤1.301234196	77.19	64.2 - 87.3	60.00	45.2 - 73.6	1.93	1.3 - 2.8	0.38	0.2 - 0.6

O

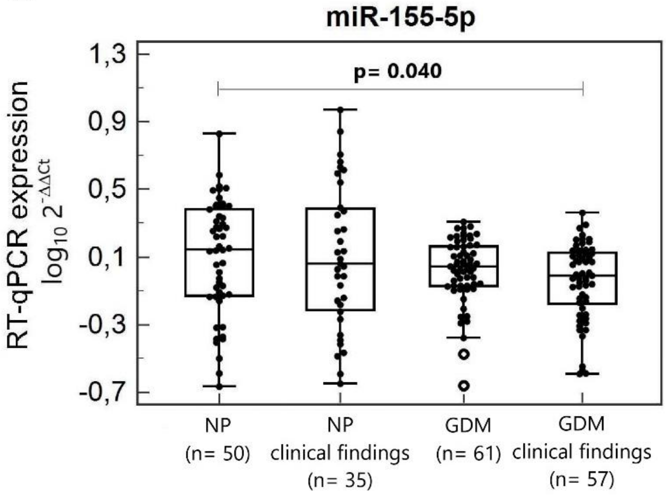

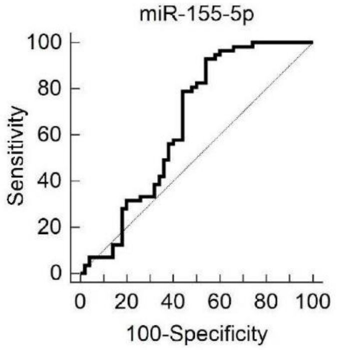

Area under the ROC curve (AUC)	0.651
Standard Error	0.0568
95% Confidence interval	0.553 to 0.740
Significance level P (Area=0.5)	0.0078

Estimated sensitivity at fixed specificity			
Specificity	Sensitivity	95% CI	Criterion
90.00	7.02	1.75 to 33.33	≤0.41116858

Criterion	Sensitivity	95% CI	Specificity	95% CI	+LR	95% CI	-LR	95% CI
≤1.599882473	92.98	83.0 - 98.1	46.00	31.8 - 60.7	1.72	1.3 - 2.2	0.15	0.06 - 0.4

P

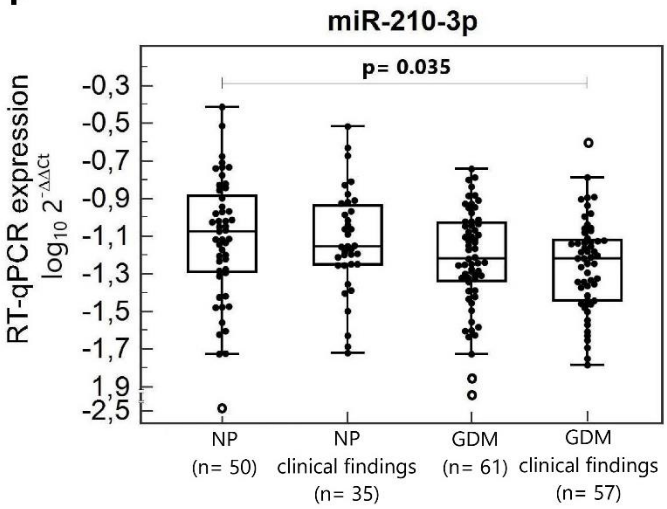

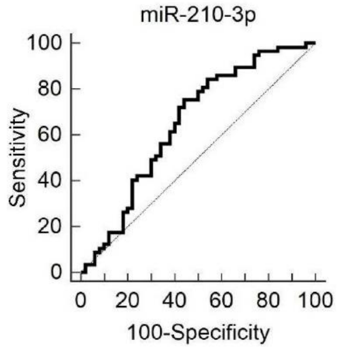

Area under the ROC curve (AUC)	0.650
Standard Error	0.0549
95% Confidence interval	0.552 to 0.740
Significance level P (Area=0.5)	0.0063

Estimated sensitivity at fixed specificity			
Specificity	Sensitivity	95% CI	Criterion
90.00	12.28	1.75 to 31.58	≤0.02669506

Criterion	Sensitivity	95% CI	Specificity	95% CI	+LR	95% CI	-LR	95% CI
≤0.07453648	75.44	62.2 - 85.9	56.00	41.3 - 70.0	1.71	1.2 - 2.4	0.44	0.3 - 0.7

Supplementary Figure S5: Aberrant microRNA expression profile in children descending from GDM complicated pregnancies with abnormal clinical findings**.** (**A–M**) Up-regulation of miR-17-5p, miR-20a-5p, miR-20b-5p, miR-21-5p, miR-29a-3p, miR-103a-3p, miR-126-3p, miR-133a-3p, miR-143-3p, miR-181a-5p, miR-195-5p, miR-221-3p, and miR-499a-5p and (**N–P**) down-regulation of miR-92a-3p, miR-155-5p, and miR-210-3p was observed in children descending from GDM complicated pregnancies with abnormal clinical findings, when the comparison to the controls with normal clinical findings was performed. NP, normal pregnancies; GDM, gestational diabetes mellitus.

Supplementary Table S1: A list of predicted targets of appropriate microRNAs dysregulated in whole peripheral blood of children descending from GDM complicated pregnancies in relation to obesity using miRWalk2.0 database (putative microRNA binding sites predicted by miRWalk algorithm within mRNA selected regions).

microRNA	Predicted targets - OBESITY
miR-1	ACSM3, BDNF, CCL2, EDN1, FABP2, MCHR1, NR3C1, PTPN1, TTR
miR-16-5p	ADRBK1, ALOX12, APLN, APOC3, ATXN2, CCDC80, FASN, HNF1A, HTR2A, MAF, RNF216
miR-17-5p	ADIPOR2, ALOX15, ANGPTL4, ANKK1, CTSK, CTSS, FAIM2, FAM71F1, FGFR1, GAD2, GCKR, GFPT1, GIPR, HIF1A, HK1, HTR2A, IAPP, IL10, IRX3, LEP, LPIN1, MCHR2, MMP2, MTHFR, MTMR9, NR1H3, PCK1, PNPLA1, PNPLA2, PNPLA3, PPARA, PPARD, PPARGC1B, RNF216, SELP, SERPINE1, SLC6A4, SPP1, SREBF1, SSTR2, STAT3, TNFRSF1B, TWIST1
miR-20a-5p	ABCA1, CLOCK, CRTC3, ESR1, HTR2A, IL8, MAP3K5, MCHR2, MFN2, MMP3, NFE2L2, NOS1AP, NPC1, PCK1, PFKP, PNPLA4, SLC2A4, TRPV1, TTR, UCP3
miR-20b-5p	ADIPOR2, ALOX15, ANGPTL4, ANKK1, CTSK, CTSS, FAIM2, FAM71F1, FGFR1, GAD2, GCKR, GFPT1, GIPR, HIF1A, HK1, HTR2A, IAPP, IL10, IRX3, LEP, LPIN1, MCHR2, MMP2, MTMR9, NR1H3, PCK1, PNPLA1, PNPLA3, PPARA, PPARD, PPARGC1B, RNF216, SELP, SERPINE1, SLC6A4, SPP1, SREBF1, SSTR2, STAT3, TNFRSF1B, TWIST1
miR-21-5p	GFPT1, LITAF, MSTN, PPARA, TLR4,
miR-23a-3p	ABCA1, ADRBK1, APOA5, CLOCK, CTSK, CTSS, F8, FASN, HSD11B1, IGF1, IL8, IRS2, LITAF, LPIN1, MAP3K5, NPC1, PNPLA1, SAA2, SLC6A14, SYT4, TLR4
miR-26a-5p	ADCY3, ADM, CCL13, CCL2, CNR1, CRP, ESR1, FAM71F1, HGF, IL6, INHBB, MTTP, NAMPT, PON1, PPARGC1B, SELP, SYT4,
miR-29a-3p	DGAT1, IGF1, LEP, LPL, SIRT1, SLC6A14, TNFRSF1A
miR-92a-3p	ADCY3, ADRB1, CCL13, CIDEA, CST3, DOK5, GHSR, GNB3, NPC1, OPRM1, PCK1, RORA, STEAP4, TWIST1
miR-100-5p	PPARGC1B, SLC27A4
miR-103a-3p	ADRB2, ALOX12, APLN, ATXN2, BDNF, CRP, DLK1, FASN, FDFT1, GPER1, HSPA1B, HTR2A, IRS2, ITGAX, KLF7, LIPE, MFN2, MMP3, MTMR9, PHB, TBC1D1, TNMD, UCP2
miR-125b-5p	ADRBK1, CEBPA, DGAT1, ENPP1, GNB3, HTR6, IGFBP3, LEP, MMP2, PLA2G16, PNPLA1, PNPLA5, STAT3, TBC1D1, VDR
miR-126-3p	APOA5
miR-133a-3p	GALR1, GM2A, MMP14, SLC27A4
miR-143-3p	ADRB2, AZGP1, BMPR1A, CHRNA2, ENPP1, FAM71F1, GNB3, GOT1, HTR2C, IL18, LPIN1, NQO1, NTRK2, NUAK2, PPARD, SERPINE1, SLC27A4, SREBF1, TUB
miR-146a-5p	ADAM17, GAD2, NMU, NTRK2, PTPN1, SCD, TMEM18
miR-155-5p	CEBPB, CETP, GNAS, GOT1, IAPP, INSIG2, MAP3K5, NR1H3, OPRM1, PMCH, TCF7L2
miR-181a-5p	AQP9, AR, CIDEA, EGFL6, F11R, FDFT1, IL1A, ITLN1, LEPR, LMNA, MAOA, MAPK1, NOS1AP, NOS3, NPFFR2, NR3C1, PCSK1, PRKAA1, SIRT1, STEAP4, TBC1D1, TCF7L2, TWIST1, WNT5B
miR-195-5p	ADRBK1, ALOX12, APLN, APOC3, ATXN2, CCDC80, FASN, HNF1A, HTR2A, MAF, RNF216
miR-199a-5p	ADIPOR1, CAPN3, CEBPB, CLOCK, CRTC3, CXCL5, F11R, FAM71F1, GGT1, HTR2A, IGFBP3, IL6, IRS1, LMNA, PCK1, RNF216, SLC25A13, SLC27A4, SLC2A4, TNF, TNMD, TRIB3, TRPV1, UBL5, VCAM1
miR-210-3p	FASN, GJA4, INHBB, ITGAX, TWIST1
miR-221-3p	ATF3, BBS4, CNR1, MTR, PCSK1, SORT1, VCAM1, XBP1
miR-499a-5p	GCG, KLF7, MAF, NR3C1, NRXN3, SCG5
miR-574-3p	CIDEA, HK1, LEP, MMP3, PPARGC1B

Supplementary Table S2: A list of predicted targets of appropriate microRNAs dysregulated in whole peripheral blood of children descending from GDM complicated pregnancies in relation to hypertension using miRWalk2.0 database (putative microRNA binding sites predicted by miRWalk algorithm within mRNA selected regions).

microRNA	Predicted targets - HYPERTENSION

miR-1	ACSM3, ARG2, BDNF, CCL2, DRD1, EDN1, KCNA5, KCNMA1, LTA, MEX3C, NR3C1, PTPN1, SLC6A19, SLC8A1, SLCO1B1, TTR
miR-16-5p	ALOX12, APLN, ATP2A2, CTH, CXCL10, GHR, HTR2A, KL, NISCH, P2RY2, SGK1, SLC12A2, VEGFA, XDH
miR-17-5p	ACVRL1, ADORA2B, ADRA1A, APEX1, ATP2A2, ATP2B1, BMP7, BMPR2, CASP8, CD36, CX3CL1, CYP4F2, ECE1, FLT1, GSTM3, HIF1A, HTR2A, IAPP, IL10, IL23R, KCNMA1, KDR, KLC1, KYNU, LEP, MMP2, MTHFR, NOS1, NOX4, OPTN, PPARA, PRCP, PTGIS, RGS2, ROCK2, SELP, SERPINE1, SLC22A3, SLC26A4, SLC2A5, SLC6A4, SLC6A9, SMAD5, SREBF1, STAT3, TNFRSF1B, TRPM6, VEGFA, XDH
miR-20a-5p	ABCA1, ATP1A2, CD36, CYP3A5, DRD1, ERAP1, ESR1, F3, FLT1, FMO3, GUCY1A3, HTR2A, MFN2, MMP3, NOX1, RLN1, SLC12A3, SLC22A2, SMAD1, TGFBR2, TRPC4, TTR
miR-20b-5p	ACVRL1, ADORA2B, ADRA1A, APEX1, ATP2A2, ATP2B1, BMP7, BMPR2, CASP8, CD36, CX3CL1, CYP4F2, ECE1, FLT1, GSTM3, HIF1A, HTR2A, IAPP, IL10, IL23R, KCNMA1, KDR, KLC1, KYNU, LEP, MMP2, MTHFR, NOS1, NOX4, OPTN, PPARA, PRCP, PTGIS, RGS2, ROCK2, SELP, SERPINE1, SLC22A3, SLC26A4, SLC2A5, SLC6A4, SLC6A9, SMAD5, SREBF1, STAT3, TNFRSF1B, TRPM6, VEGFA, XDH
miR-21-5p	CXCL10, EDNRB, KLF5, PPARA, THPO
miR-23a-3p	ABCA1, ADRA2B, ARG1, ATP1A1, CXCL12, EDNRB, HSD11B1, IGF1, KLF5, MAPK14, MBL2, PRCP, TRPM6, ZNF652
miR-26a-5p	ADM, ATP1A2, CALCRL, CCL2, CTH, ENG, ESR1, FLT1, HGF, HLA-A, IER3, IL6, NOS1, PCNA, PIM1, PON1, PTGS2, PTX3, ROBO4, SELP, SLC12A2, SLCO4C1, SMAD1, STK39, TAP1, TRPC3, TRPC4, WNK1, XDH, ZNF652
miR-29a-3p	APLNR, CASP8, CXCL10, IGF1, KNG1, LEP, LPL, NOS2, SDK1, SGK1, VEGFA, XPNPEP1
miR-92a-3p	ADRB1, ATP2A2, CCR2, CHGA, CST3, CYBA, GNB3, GSTA1, IL23R, NOX4, PEPD, PIK3R1, SLC9A1, STEAP4, TRPC4
miR-103a-3p	ADRA1A, ADRB2, ALAD, ALOX12, APLN, BDNF, CD40, CHGA, CRP, CYP2C9, DRD1, EDNRB, FURIN, HSPA1B, HTR2A, KLC1, LIPE, MFN2, MLYCD, MMP3, NISCH, NOS1, RNLS, ROBO4, SLC22A2, SLC6A2, SLC7A1, SLCO4C1, TGFBR3, UCP2, UMOD, VWF, XDH
miR-125b-5p	ADRA1A, ANPEP, BDKRB2, BMPR1B, CCR2, CCR5, CYP11B2, EMILIN1, ENPEP, ENPP1, EPO, GNB3, KCNK3, LEP, MLYCD, MMP2, PSMB9, ROBO4, STAT3
miR-133a-3p	ADRA2B, ALAD, CHGB, CXCL12, GOSR2, KCNMA1, KYNU, MAPK14, PEPD, SCG2, SLC22A2
miR-143-3p	ADRB2, ARHGEF1, CCR2, CD40, CYP11B1, CYP1A2, CYP2C9, ENPP1, EPO, FGB, GHR, GNB3, IER3, IL18, KLF5, NFKBIL1, PDE5A, SERPINC1, SERPINE1, SLC12A3, SREBF1, UMOD, VNN1, ZNF652
miR-146a-5p	CAT, CFH, CYP11A1, HSPA1A, INPPL1, KCNK3, KCNMA1, NOS1, NOX4, PRCP, PTGS2, PTPN1, RHOA, THPO, WISP1, WNK4, ZNF652
miR-155-5p	ADRA1A, ARG1, CD36, CETP, DRD1, GNAS, IAPP, NPPA, SMAD1, ZNF652
miR-181a-5p	AR, ATP1B1, ATP2B1, AVPR1A, CACNB2, CASP8, CTH, CYP4F2, EDNRA, F11R, F3, GREM1, HSD3B1, IL1A, KCNMA1, KL, LEPR, MAOA, MAPK1, NOS3, NR3C1, PTGS2, SLC7A1, SOD3, STEAP4, TGFBR2, VIP, WISP1, ZNF652
miR-195-5p	ALOX12, APLN, ATP2A2, CTH, CXCL10, GHR, HTR2A, KL, NISCH, P2RY2, SGK1, SLC12A2, VEGFA, XDH
miR-199a-5p	CCR2, CHGA, CSK, DDAH2, F11R, GGT1, HTR2A, IL6, MAT1A, MMP9, PTGIS, SCG2, TNC, TNF, VCAM1
miR-210-3p	ATP2A2, BDKRB2, IER3, NR3C2
miR-221-3p	ATP1A1, CD36, KLC1, MTR, RNLS, VCAM1
miR-499a-5p	CACNB2, CLU, CYP3A4, FLT1, GUCY1A3, NR3C1, SLC12A2, SLC22A8
miR-574-3p	ID2, LEP, MMP3, NISCH, ZNF652

Supplementary Table S3: A list of predicted targets of appropriate microRNAs dysregulated in whole peripheral blood of children descending from GDM complicated pregnancies in relation to glucose intolerance using miRWalk2.0 database (putative microRNA binding sites predicted by miRWalk algorithm within mRNA selected regions).

microRNA	Predicted targets – GLUCOSE INTOLERANCE
miR-16-5p	HNF1A
miR-17-5p	CD36, HFE, PPARD, TNFRSF1B
miR-20a-5p	CD36, SLC2A4
miR-20b-5p	CD36, HFE, PPARD, TNFRSF1B
miR-23a-3p	HSD11B1
miR-26a-5p	NAMPT
miR-29a-3p	NEUROG3, TNFRSF1A
miR-103a-3p	NEUROG3, PRKCZ, TNMD
miR-125b-5p	ENPP1
miR-143-3p	ENPP1, GOT1, PPARD
miR-146a-5p	CFH, INPPL1
miR-155-5p	CD36, GOT1, TCF7L2
miR-181a-5p	NOS3, TCF7L2
miR-195-5p	HNF1A
miR-199a-5p	GGT1, SLC2A4, TNMD
miR-221-3p	CD36
miR-499a-5p	GCG, SCG5
miR-574-3p	AGER, PRKCZ

Supplementary Table S4: A list of predicted targets of appropriate microRNAs dysregulated in whole peripheral blood of children descending from GDM complicated pregnancies in relation to lipid metabolism disease using miRWalk2.0 database (putative microRNA binding sites predicted by miRWalk algorithm within mRNA selected regions).

microRNA	Predicted targets – LIPID METABOLISM DISEASE
miR-17-5p	PNPLA2
miR-20b-5p	PNPLA2

Supplementary Table S5: A list of predicted targets of appropriate microRNAs dysregulated in whole peripheral blood of children descending from GDM complicated pregnancies in relation to type 2 diabetes using miRWalk2.0 database (putative microRNA binding sites predicted by miRWalk algorithm within mRNA selected regions).

microRNA	Predicted targets – TYPE 2 DIABETES
miR-1	CD28
miR-16-5p	FASN
miR-17-5p	CD28, CD36, FXN, PPARA, SERPINE1
miR-20a-5p	CD36
miR-20b-5p	CD28, CD36, FXN, PPARA, SERPINE1
miR-21-5p	PPARA
miR-23a-3p	FASN
miR-26a-5p	NAMPT
miR-103a-3p	FASN, VWF
miR-133a-3p	CD28
miR-143-3p	CD28, SERPINE1, FXN
miR-155-5p	CD36
miR-181a-5p	LEPR
miR-195-5p	FASN
miR-210-3p	FASN
miR-221-3p	CD36

Supplementary Table S6: A list of predicted targets of appropriate microRNAs dysregulated in whole peripheral blood of children descending from GDM complicated pregnancies in relation to heart septal defect using miRWalk2.0 database (putative microRNA binding sites predicted by miRWalk algorithm within mRNA selected regions).

microRNA	Predicted targets – HEART SEPTAL DEFECT
miR-16-5p	VEGFA
miR-17-5p	BMPR2, MTHFR, VEGFA
miR-20b-5p	BMPR2, MTHFR, VEGFA
miR-21-5p	RTN4
miR-29a-3p	TLL1, VEGFA
miR-92a-3p	ACTC1
miR-143-3p	CRELD1
miR-155-5p	NPPA, PTPN11
miR-181a-5p	TLL1
miR-195-5p	VEGFA

Supplementary Table S7: A list of predicted targets of appropriate microRNAs dysregulated in whole peripheral blood of children descending from GDM complicated pregnancies in relation to heart valve disease using miRWalk2.0 database (putative microRNA binding sites predicted by miRWalk algorithm within mRNA selected regions).

microRNA	Predicted targets – HEART VALVE DISEASE
miR-133a-3p	VKORC1

Supplementary Table S8: A list of predicted targets of appropriate microRNAs dysregulated in whole peripheral blood of children descending from GDM complicated pregnancies in relation to heart disease using miRWalk2.0 database (putative microRNA binding sites predicted by miRWalk algorithm within mRNA selected regions).

microRNA	Predicted targets – HEART DISEASE
miR-1	CCL2, CDKN2B, CNDP1, CPB2, EDN1, ENTPD1, FN1, LTA, MMP8, SELPLG
miR-16-5p	APOC3, CXCL10, GHR, HNF1A, HTR4, PTH, VEGFA
miR-17-5p	ANGPTL4, ATXN1, CD36, CX3CL1, HFE, HIF1A, IL10, IL1R1, KDR, LDLR, LEP, LIMS1, MMP2, MTHFR, NR1H3, PPARA, SELP, SERPINE1, VEGFA
miR-20a-5p	ABCA1, CD36, ESR1, F3, HSPA8, IL8, LIMS1, MMP3, MTHFD1L, NPC1
miR-20b-5p	ANGPTL4, ATXN1, CD36, CX3CL1, HFE, HIF1A, IL10, IL1R1, KDR, LDLR, LEP, LIMS1, MMP2, MTHFR, NR1H3, PPARA, SELP, SERPINE1, VEGFA
miR-21-5p	CXCL10, EDNRB, PPARA, TLR4
miR-23a-3p	ABCA1, APOA5, CBS, EDNRB, ENTPD1, FCAR, IFNG, IGF1, IL8, MAPK14, MBL2, NPC1, SELPLG, TLR4
miR-26a-5p	CBS, CCL2, CRP, ESR1, FCAR, HSPA8, HTR4, IFNG, IL6, MTTP, PON1, SELP
miR-29a-3p	CXCL10, FCGR2A, IGF1, LEP, VEGFA
miR-92a-3p	CCR2, CNDP1, CPE, CYBA, DUSP1, GP6, IFNG, KAT2B, NPC1
miR-100-5p	CXCL16
miR-103a-3p	CRP, CYP2C9, EDNRB, HTR4, MMP3, NOD1, PTH, SELPLG, VAMP8, VWF
miR-125b-5p	ATXN1, CCR2, CCR5, CEBPA, ENPP1, EPO, IGFBP3, LBH, LEP, MMP2, SCARB1, SELPLG, TNFSF4, VAMP8
miR-126-3p	APOA5
miR-133a-3p	CPB2, FN1, KCND3, MAPK14, MMP14, SUMO1, VKORC1
miR-143-3p	ALB, CCR2, CPB2, CYP2C9, ENPP1, EPO, GHR, IFNG, IL18, KCND3, MGP, NFATC1, NQO1, SELL, SERPINE1
miR-146a-5p	ALOX5AP, CFH, ENTPD1, TFAP2B
miR-155-5p	CD36, CETP, NR1H3, PTPN11, TCF7L2

miR-181a-5p	EDNRA, F3, IL1A, IL1R1, KAT2B, LMNA, MMP8, NOS3, PON3, SOD3, TCF7L2
miR-195-5p	APOC3, CXCL10, GHR, HNF1A, HTR4, PTH, VEGFA
miR-199a-5p	CCR2, CXCL16, CXCR6, GGT1, GP6, HTR4, IGFBP3, IL6, LMNA, MMP9, TNC, TNF
miR-221-3p	CD36, LIMS1
miR-499a-5p	CYP3A4, ENTPD1, HSPA8, NPC1L1
miR-574-3p	AGER, LEP, MMP3, NFATC1

Supplementary Table S9: A list of predicted targets of appropriate microRNAs dysregulated in whole peripheral blood of children descending from GDM complicated pregnancies in relation to heart failure using miRWalk2.0 database (putative microRNA binding sites predicted by miRWalk algorithm within mRNA selected regions).

**microRNA**	**Predicted targets – HEART FAILURE**
miR-1	EDN1, GJA1 JPH2, PPP1R1A, RAMP3, SFTPB, TTR
miR-16-5p	APLN, ATP2A2, HTR4, KCNE1, NISCH, PPP1R2, PPRC1, VEGFA
miR-17-5p	ADRA1A, ADRA1B, ATP2A2, BVES, CFLAR, CNR2, DYRK1A, ERBB2, GJA1, HFE, IL10, IL5, LEP, LRG1, MMP2, NOS1, OPA1, SLC6A4, SPP1, TPM1, UNC93B1, VEGFA
miR-20a-5p	ADRA1B, F3, HDAC4, PTHLH, SLC2A4, STC1, TTR, ZFPM2
miR-20b-5p	ADRA1A, ADRA1B, ATP2A2, BVES, CFLAR, CNR2, DYRK1A, ERBB2, GJA1, HFE, IL10, IL5, LEP, LRG1, MMP2, NOS1, OPA1, SLC6A4, SPP1, TPM1, UNC93B1, VEGFA
miR-21-5p	CFLAR, EDNRB, IL9, KCNH2, LAMA4, RTN4, TLR4, TPM1
miR-23a-3p	CFLAR,EDNRB, FOXP1, IFNG, IGF1, MAP4, MAPK14, MBL2, NRG1, PROM1, RAMP3, RYR2, TLR4
miR-26a-5p	ADM, CALCRL, CRP, FRMD4B, HGF, HTR4, IFNG, IL6, NOS1, NRG1, SFTPB, TRPC3, UGT1A1
miR-29a-3p	BDKRB1, IGF1, IL1RL1, LEP, TNFRSF1A, TPM1, VEGFA
miR-92a-3p	ADRB1, ATP2A2, CCR2, CHGA, CST3, CYBA, DUSP1, GNB3, GRK5, IFNG, IL1RL1, JPH2, RAPGEF3, SLC9A1
miR-100-5p	CXCL16
miR-103a-3p	ADRA1A, ADRA1B, ADRB2, APLN, CHGA, CRP, CYP27B1, EDNRB, ESRRA, FKBP1B, HSPA1B, HTR4, MAP4, NISCH, NOL3, NOS1, NPR2, NRG1, PTHLH, SFTPB, UCP2, VWF
miR-125b-5p	ADRA1A, BSG, CCR2, CD34, CEBPA, CYP11B2, ERBB2, ESRRA, GNB3, LAMA4, LEP, MMP2, MYL9
miR-126-3p	KCNE1
miR-133a-3p	CYP27B1, FOXC1, KCNE1, MAPK14, PARP1
miR-143-3p	ADRB2, CCR2, CNR2, FOXO1, GATM, GNB3, IFNG, IL18, PDE5A, SFTPB
miR-146a-5p	BSG, CYP27B1, NOS1, PPP1R1A, STC1, TPM1
miR-155-5p	ADRA1A, GATM, NPPA, NRG1, PDK1
miR-181a-5p	EDNRA, F3, FOXP1, GATM, LEPR, LMNA, NOS3, PPP1R2, SOD3
miR-195-5p	APLN, ATP2A2, HTR4, KCNE1, NISCH, PPP1R2, PPRC1, VEGFA
miR-199a-5p	CCR2, CHGA, CXCL16, ERBB2, FKBP1B, HTR4, IL6, KCNH2, KLF15, LMNA, LRG1, MAP4, MMP9, PPP1R2, SFTPB, SLC2A4, TNF, VCAM1
miR-210-3p	ATP2A2
miR-221-3p	PPP1R2, THBS1, TIMP4, VCAM1, ZFPM2
miR-499a-5p	IL1RL1, TIMP4
miR-574-3p	AGER, LEP, NISCH, SRF

Supplementary Table S10: A list of predicted targets of appropriate microRNAs dysregulated in whole peripheral blood of children descending from GDM complicated pregnancies in relation to venous insufficiency using miRWalk2.0 database (putative microRNA binding sites predicted by miRWalk algorithm within mRNA selected regions).

microRNA	Predicted targets – VENOUS INSUFFICIENCY
miR-1	P2RX5
miR-17-5p	CCL5, P2RX5, SERPINE1
miR-20b-5p	CCL5, P2RX5, SERPINE1
miR-29a-3p	P2RX5
miR-100-5p	P2RX5
miR-125b-5p	P2RX5
miR-143-3p	SERPINE1
miR-146a-5p	CCL5
miR-199a-5p	P2RX5

Supplementary Table S11: A list of predicted targets of appropriate microRNAs dysregulated in whole peripheral blood of children descending from GDM complicated pregnancies in relation to pulmonary embolism using miRWalk2.0 database (putative microRNA binding sites predicted by miRWalk algorithm within mRNA selected regions).

microRNA	Predicted targets – PULMONARY EMBOLISM
miR-1	CPB2
miR-17-5p	MTHFR
miR-20a-5p	F3
miR-20b-5p	MTHFR
miR-26a-5p	CRP
miR-103a-3p	CRP
miR-133a-3p	CPB2
miR-143-3p	ALB, CPB2
miR-155-5p	NPPA
miR-181a-5p	F3
miR-199a-5p	SERPINF2
